# Molecular basis for differential PIP_2_-mediated association between vinculin and its splice isoform metavinculin

**DOI:** 10.1016/j.jbc.2025.110232

**Published:** 2025-05-14

**Authors:** Mohammad Ashhar I. Khan, Venkat R. Chirasani, Muzaddid Sarker, Laura McCormick, Sharon L. Campbell

**Affiliations:** 1 Department of Biochemistry & Biophysics, University of North Carolina at Chapel Hill, Chapel Hill, North Carolina, USA; 2 R. L. Juliano Structural Bioinformatics Core, University of North Carolina at Chapel Hill, Chapel Hill, North Carolina, USA; 3 Lineberger Comprehensive Cancer Center, University of North Carolina at Chapel Hill, Chapel Hill, North Carolina, USA

**Keywords:** actin, cardiomyopathy, heart, membrane, metavinculin, vinculin, phosphatidylinositol 4,5-bisphosphate--PIP_2_

## Abstract

Vinculin (Vcn) and its splice variant metavinculin (MVcn) are cell adhesion proteins that regulate cell morphology, adhesion, and motility. They function as scaffold proteins that anchor membrane receptors to filamentous actin (F-actin) at focal adhesions and cell–cell junctions. MVcn bears an extra 68 amino acid insert in the tail domain and is selectively expressed in cardiac and smooth muscle cells at substoichiometric levels relative to Vcn. Mutations in the MVcn tail domain (MVt) promote cardiomyopathy, yet how these mutations alter ligand interactions to promote defects in force transduction and reduced blood flow is unclear. One difference between Vcn and MVcn lies in the ability to reorganize F-actin, with MVcn negatively regulating Vcn-mediated F-actin bundling. Vcn associates with phosphatidylinositol 4,5-bisphosphate (PIP_2_) through its tail domain (Vt) to drive recruitment, activation, and focal adhesion turnover. However, it remains unclear whether MVcn specifically associates with PIP_2_-containing membranes and how such interactions might influence its functional interplay with Vcn in tissues where both isoforms coexist. To evaluate the interaction of MVt and MVt cardiomyopathy mutants with PIP_2_ membranes in comparison with Vt, we conducted mutagenesis, phospholipid-association assays, and computational modeling. We found that MVt shows reduced association for PIP_2_-containing liposomes relative to Vt due to sequence differences within the insert region. Moreover, mutations in MVt that promote cardiomyopathies do not affect PIP_2_-dependent lipid association. These findings suggest that MVcn differs from Vcn in driving PIP_2_-mediated membrane association and sheds light on the coordinate role of Vcn and MVcn in membrane association as well as MVcn cardiomyopathy defects.

Vinculin (Vcn) and metavinculin (MVcn) are cell adhesion proteins that are fundamental to the proper functioning of cells, affecting their ability to grow, move, and differentiate. Their coordinated activity is essential for maintaining cellular and tissue integrity (1, 2, 3, 4). Vcn and MVcn localize to focal adhesions (FA) and cell–cell junctions, where they function as scaffolds that connect cell surface receptors to the actin cytoskeleton (1, 5, 6, 7). While Vcn is ubiquitously expressed, MVcn is selectively expressed in muscle cells at substoichiometric levels relative to Vcn (8, 9).

Vcn and MVcn can exist in active and inactive states. In the inactive state, ligand interactions are obscured due to autoinhibitory contacts between the head and tail domains (10) with activation required to promote scaffolding (1, 11, 12). The tail domain of Vcn (Vt) binds filamentous actin (F-actin) and acidic phospholipids and is comprised of an N-terminal strap (N-strap), five helices (forming a helix bundle), and a C-terminal hairpin loop (13, 14) (Fig. 1*A*). Vcn also interacts with phosphatidylinositol 4, 5-bisphosphate (PIP_2_) through its tail domain to facilitate Vcn membrane localization, FA formation, as well as Vcn activation and turnover at FAs (15, 16, 17). Although Vcn and MVcn have identical head domains, MVcn contains a 68 amino acid insert in the C-terminal tail domain (18). The MVcn tail domain (MVt) retains the helix bundle architecture, but the strap 1′ and helix 1′ within the insert replace the strap and helix 1 of Vcn (Fig. 1*A*) (19, 20).

**Figure 1:**
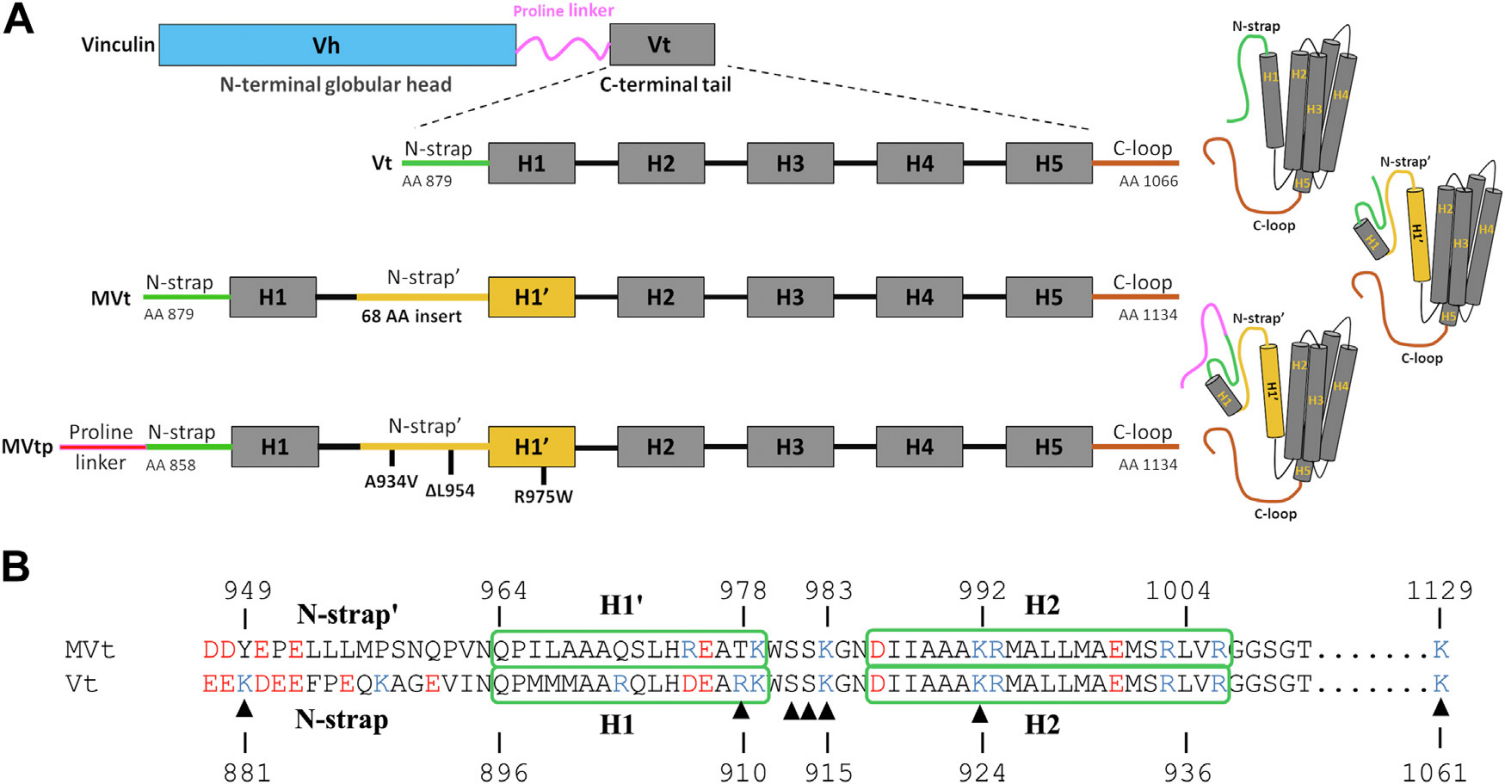
**Vinculin and metavinculin tail constructs and sequence differences.***A*, domain map of full-length vinculin and metavinculin tail constructs (Vt, MVt and MVtp) used in the study. The 68 amino acid insert in MVt is shown in *yellow*. Locations of three cardiomyopathy (CM) mutations are denoted on the MVtp construct which contains part of the proline-rich region. *B*, sequence alignment of the N-terminal region comparing MVt and Vt. Residues within the basic collar region consisting of the Strap/Strap′, H1/H1′, and H2 that interact with PIP_2_ are annotated with a triangle symbol. Two out of nine basic residues (K881 and R910) that are predicted to be critical for Vt-PIP_2_ head group recognition are not conserved in MVt. The basic and the acidic residues in the Strap/H1 (Vt) and in Strap′/H1′ (MVt) are colored in *red* and *blue*, respectively.

One key difference between Vcn and MVcn lies in the ability to reorganize F-actin filaments. Vcn induces bundling of actin filaments *via* the tail domain to promote adhesion, regulate motility, and confer mechanosensitivity, whereas MVcn retains F-actin binding but cannot bundle actin filaments (21, 22, 23). Moreover, MVt can inhibit Vt actin-bundling activity, even at low MVt:Vt ratios (24). These findings led to our hypothesis that MVcn negatively regulates Vcn reorganization of F-actin into bundles (24, 25). However, it is unclear whether MVcn similarly associates with membranes in a PIP_2_-dependent manner to regulate MVcn function.

Deregulation of MVcn and Vcn is associated with debilitating heart conditions such as dilated cardiomyopathy (DCM) and hypertrophic cardiomyopathy, which are leading causes of congestive heart failure and arrhythmias (26, 27, 28, 29). Patients with DCM possess an enlarged heart due to the dilation or stretching of the heart muscle, leading to inefficient blood circulation believed to arise from compromised contractile properties (30, 31). Notably, mutations in the MVcn insert (A934V, R975W, ΔL954) (Fig. 1*A*), which were first identified in patients with cardiomyopathy (CM), promote disordered F-actin assemblies (24), disorganized costameres (26) (cell-matrix), and intercalated discs (cell–cell junctions) (27, 32). These findings led to our hypothesis that MVcn negatively regulates Vcn function in muscle cells, with dysregulation altering coordinate regulation of the isoforms (24, 25). However, it is unclear whether differences in PIP_2_-mediated membrane association between MVcn and Vcn contribute to altered cell morphology and improper force generation associated with CMs.

To better understand how Vcn recognizes PIP_2_ and inserts into the membrane, we previously generated an experimentally supported structural model of Vt in complex in large unilamellar vesicles (LUVs)/liposomes containing PIP_2_ and identified two patches of basic amino acids within the Vt basic collar and basic ladder that mediate phospholipid association (33, 34). According to this structural model, the basic collar region specifically interacts with the PIP_2_ head group and is composed of basic residues from helices 1 to 2 and the C-terminal loop (Fig. 1*B*), whereas the basic ladder region comprises basic residues within helices 3 to 4 that facilitate acidic phospholipid insertion into the lipid bilayer. A recent coarse-grained model of Vt/PIP_2_ using a PIP_2_-C_18_ membrane mimetic provides additional support for this structural model (35). While a crystal structure of a Vt mutant in complex with a soluble short-chain PIP_2_ derivative (PIP_2_-C_8_) has been reported (17), Vt was reported to oligomerize as a trimer complexed to PIP_2_-C_8_ in a manner that is inconsistent with membrane insertion, possibly due to the use of a Vt mutation in the context of a short-chain PIP_2_ derivative (17). Moreover, NMR analyses of Vt in the presence of PIP_2_-C_8_ indicated that this short-chain PIP_2_ derivative does not interact with the key head group interaction site in the basic collar or promote Vt oligomerization (34). A crystal structure of MVt complex with a similar short-tail, soluble PIP_2_ derivative has also been obtained (PDB: 5L0C) (36). In this structure, MVt is arranged in a symmetric domain-swapped dimer that bridges two short-chain PIP_2_ molecules. Although the crystal structure reported by Chinthalapudi *et al.* (2016) provides a preliminary model for MVt–PIP_2_ interactions, these studies were performed using a soluble short-chain PIP_2_ which limits the generalization of the model to biologically relevant membranes. For these reasons, we used the reported model of Vt in complex with full-length PIP_2_ embedded in physiologically relevant liposomes/LUVs as a starting point for our analyses (34).

To evaluate phospholipid interaction differences between Vt, MVt, and MVt CM mutants, we conducted structural, biochemical, and computational studies. Based on our previous structural model of Vt–PIP_2_ interactions (34), we first performed sequence analysis to identify key differences between Vt and MVt in regions critical for phospholipid association. We then experimentally validated these differences using lipid-binding assays with targeted mutations and further employed computational approaches including molecular docking and MD simulations to understand the molecular basis for the differential association behavior. We find that sequence differences in the MVt insert reduce PIP_2_ interactions. In contrast to previous observations, we find that MVt R975W mutant, as well as two other CM mutants, do not alter the specificity or the affinity of MVt for PIP_2_ liposomes. These findings provide new insights into the coordinated role of Vcn and MVcn in membrane association as well as MVcn-associated CMs.

## Results

### Vt shows higher association with PIP_2_-containing liposomes relative to MVt

Vcn associates with membranes in a PIP_2_-dependent manner *via* its tail domain (34, 37). Based on our experimentally driven structural model, we identified Vcn variants with selective impairments in PIP_2_-mediated membrane association and found that PIP_2_-mediated membrane insertion facilitates Vcn subcellular localization, activation, and FA turnover (38). While MVt shows reduced association with PIP_2_ in the context of sucrose-loaded PIP_2_ unilamellar lipid vesicles (39), it is unclear whether MVt similarly promotes phospholipid-dependent physiological membrane association. To address this question, we applied computational and experimental approaches to evaluate MVt association with liposomes of varying compositions. Our experimentally directed structural model (34) of Vt in complex with PIP_2_ embedded in physiologically relevant liposomes supports a role for the basic collar in specific recognition of the PIP_2_ headgroup. Building on this established framework, we performed sequence analysis to identify key differences between Vt and MVt in these critical binding regions (33, 34). The sequence differences in this region suggest that MVt may differentially recognize PIP_2_ relative to Vt (Fig. 1*B*).

**Figure 2:**
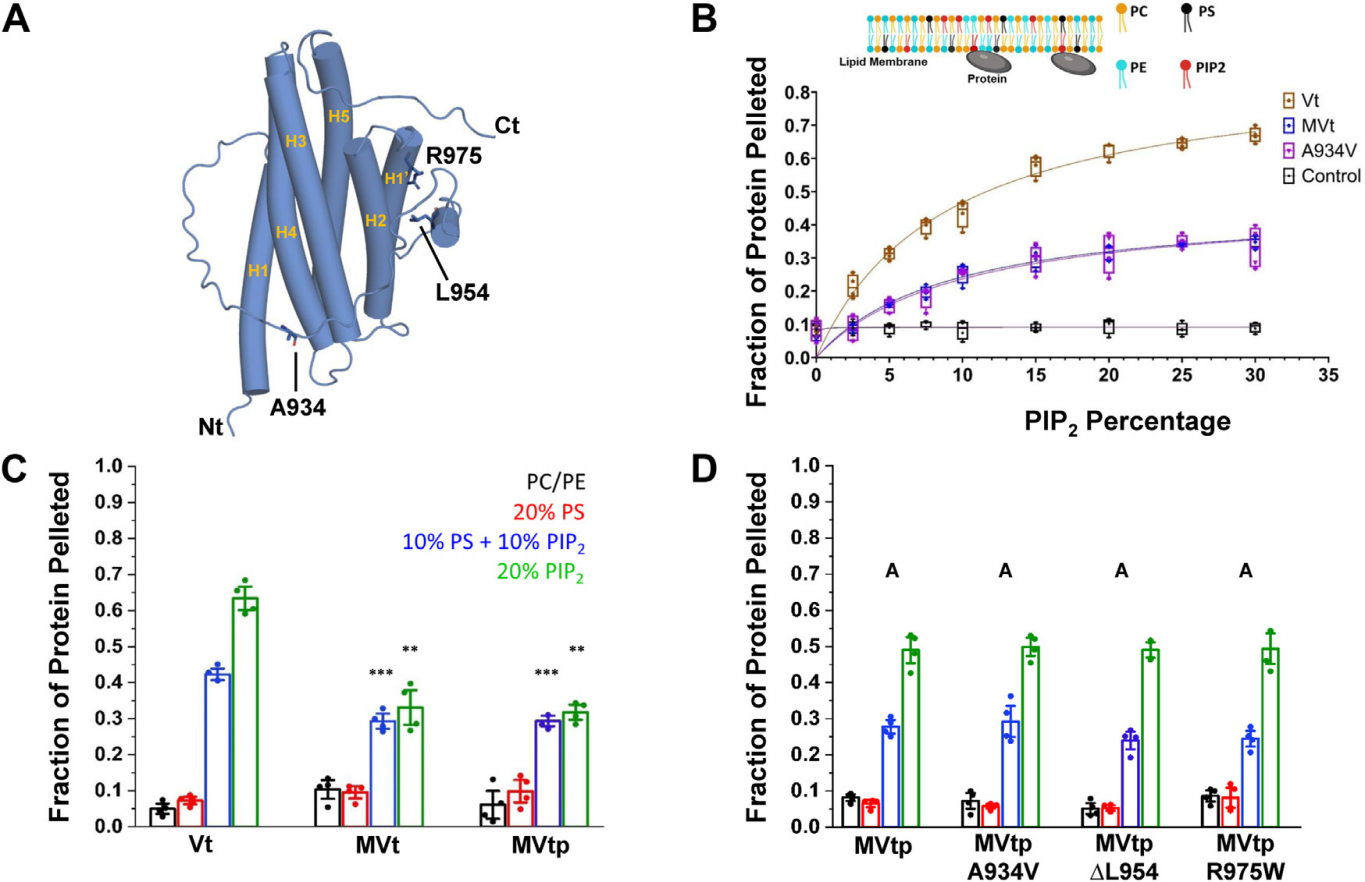
**MVt exhibits decreased PIP_2_-membrane association relative to Vt. Cardiomyopathy mutations in MVt and have minimal impact on liposome association.***A*, cartoon model of MVt with cardiomyopathy (CM)-linked residues represented as licorice. *B*, high-speed lipid cosedimentation assays examining PIP_2_ concentration-dependent membrane association of Vt and MVt. Proteins (10 μM) were incubated with LUVs containing increasing concentrations of PIP_2_ (0–30%) in a physiologically relevant membrane composition (PE/PC/PS/PIP_2_). Individual data points are represented within the box & whisker plot with bars showing range and mean. *C*, results from high speed lipid cosedimentation assays comparing Vt, MVt, or MVtp protein association with LUVs of differing composition. *D*, high speed lipid cosedimentation assays comparing the association of MVtp or MVtp CM variant proteins with LUVs of differing composition. Two-tailed *t* test comparing means of MVt and MVtp to Vt in (*D*). Binding curves were fitted using nonlinear regression to a one-site specific binding model, yielding half-maximal binding concentrations, B_max_, and binding potential (B_p_) values reported in Table 2. Error bars represent S.D. from four data points (represented as *solid* circles within the bar graph) from two independent experiments. Statistical significance was assessed using two-tailed t-tests comparing means of MVt and MVtp to Vt in (*C*). For (*D*), one-way ANOVA followed by Tukey *post hoc* test was performed for multiple comparisons of protein–PIP_2_ interaction. Means of variants that do not share the same letter (inlet alphabet) are significantly different (*D*). Statistical significance was set at: ∗∗*p* < 0.01, ∗∗∗*p* < 0.001, n.s., nonsignificant.

To experimentally assess differences in PIP_2_–membrane interaction between MVt and Vt, we conducted lipid cosedimentation assays to evaluate the apparent binding affinity (K_d_) and maximum binding capacity (B_max_) of Vt, MVt, and MVt A934V CM mutant (Fig. 2*A*) in PIP_2_ liposomes. Two types of lipid co-sed experiments were performed, protein titration with fixed PIP_2_ (10%) concentration (Fig. S1) and liposomes containing a gradient of PIP_2_ with fixed protein concentration (10 μM) (Fig. 2*B*). Protein titration experiments at fixed PIP_2_ (10%) concentration yielded apparent K_d_ values of 4.94 ± 1.56 μM for Vt and 4.66 ± 1.62 μM for MVt, with corresponding B_max_ values close to 50 for both proteins (Table 1). A protein concentration of 10 μM was used for PIP_2_ gradient experiments as it is close to the K_d_ values for both Vt and MVt (12, 33, 34, 40). The percentage of PIP_2_ in liposomes required for half-maximal binding was determined to be 8.89 ± 1.25% (0.19 ± 0.02 μM), 9.20 ± 1.80% (0.21 ± 0.03 μM), and 9.94 ± 2.52% (0.22 ± 0.05 μM) for Vt, MVt, and MVt A934V, respectively (Table 2). Lower values in this metric indicate a higher affinity for PIP_2_-enriched membranes. Notably, while the K_d_ values are similar, Vt shows > 2-fold binding potential (4.63) difference relative to MVt (2.19) and MVt A934V (2.04) (Table 2). Hence, while MVt associates with PIP_2_ in a concentration-dependent manner compared to control-charged acidic phospholipids, MVt shows an overall reduction in PIP_2_ liposome association relative to Vt (Fig. 2*B*).

**Table 1:** Apparent binding affinities (K_d_) and maximum binding capacities (B_max_) of vinculin and metavinculin tail domains in protein titration experiments at fixed PIP_2_ concentration

Protein(s)	Protein concentration (μM)	Fraction of protein pelleted
	app K_d_	B_max_
Vt	4.94 ± 1.56	0.52 ± 0.03
MVt	4.66 ± 1.62	0.42 ± 0.03

**Table 2:** Concentration of PIP_2_-containing liposomes at half-maximal binding or apparent binding affinities (K_d_), maximum binding capacities (B_max_), and binding potential (BP) of vinculin, metavinculin, and cardiomyopathy A934V variant tail domains in PIP_2_ gradient experiments

Protein(s)	PIP_2_ (%)	PIP_2_ (μM)	Fraction of protein pelleted	Binding potentials
	app K_d_	app K_d_	B_max_	B_P_
Vt	8.89 ± 1.25	0.19 ± 0.02	0.88 ± 0.04	4.63 ± 0.1
MVt	9.20 ± 1.80	0.21 ± 0.03	0.46 ± 0.03	2.19 ± 0.1
A934V	9.94 ± 2.52	0.22 ± 0.05	0.47 ± 0.04	2.04 ± 0.1

To compare Vt and MVt association with acidic phospholipid lipid vesicles of differing composition, we performed an additional series of lipid cosedimentation assays (Fig. 2*C*). Consistent with previous findings (33, 34, 39), we find that Vt shows enhanced association with PIP_2_ liposomes relative to LUVs containing phosphocholine (PC), phosphoethanolamine (PE), and 20% phospho-L-serine (PS) (Fig. 2*C*). While MVt shows some specificity for PIP_2_ liposomes over LUVs containing PC, PE, and 20% PS vesicles, association is significantly reduced compared to Vt (Fig. 2*C*), consistent with a previous report conducted in sucrose-loaded PIP_2_ containing unilamellar lipid vesicles (39). Specifically, we find that 43% of Vt is pelleted compared to 30% of MVt in 10% PS/10% PIP_2_ liposomes. Moreover, in 20% PIP_2_ liposomes, 63% of Vt is pelleted compared to 33% of MVt.

To evaluate whether MVt proline linker region plays a role in lipid association, we performed lipid cosedimentation assays using MVtp which contains an additional 21 amino acids within the proline-rich region that precedes the tail domain (Fig. 1*A*). We found that MVtp exhibits similar acidic phospholipid association and specificity for PIP_2_ liposomes compared to WT MVt (Fig. 2*C*), indicating that the additional residues within the proline linker do not influence MVt–lipid interactions. These findings taken together indicate that MVt shows reduced association with PIP_2_-containing liposomes and raises the possibility that a smaller fraction of MVcn may associate with the membrane relative to Vt.

### MVt CM mutations retain phospholipid specificity and association

Three mutations (A934V, ΔL954, R975W) that lie within the MVcn insert region have been identified in CM patients (Fig. 1*A*). While the three MVt CM mutants retain F-actin binding, we previously demonstrated that they enhance the formation of disordered actin assemblies relative to ordered stress fibers generated by Vt (24, 25). The MVt R975W CM mutant was previously reported to increase association with PIP_2_-containing small unilamellar vesicles (36), yet the A934V and ΔL954 DCM mutants have not been investigated. To better assess phospholipid association under conditions that better mimic physiological membranes, we conducted lipid cosedimentation experiments on all three CM mutants in the context of MVtp. As shown in Figure 2*D*, the CM mutants show similar cosedimentation profiles as WT MVt and MVtp in PIP_2_-containing liposomes relative to LUVs containing PC, PE, and 20% PS. These results indicate that all three MVt CM mutants retain liposome association compared to WT MVt. Based on these observations, we hypothesize that the physiological impact of MVt CM mutations results primarily from altered actin reorganization rather than lipid interactions.

### Deletion of 68-residue insert in MVt enhances PIP_2_ association

**Figure 3:**
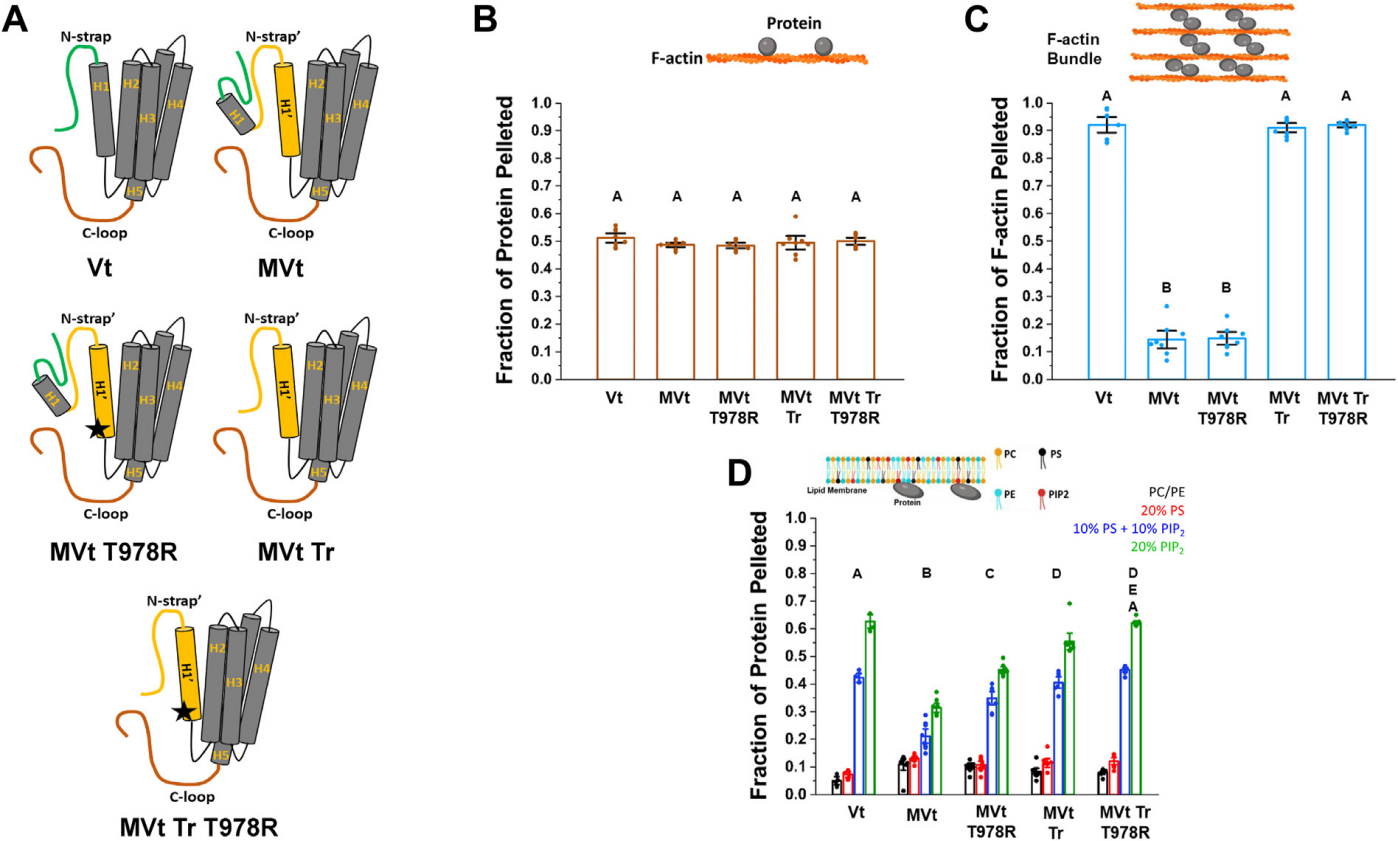
**MVt T978R substitution exhibits enhanced PIP_2_-liposome association.***A*, cartoon representation of Vt, MVt, MVt-T978R, MVt-Tr, and MVt-Tr-T978R proteins. *B*, high speed F-actin cosedimentation assays comparing F-actin binding to Vt, MVt, MVt-T978R, MVt-Tr, and MVt-Tr-T978R proteins. *C*, low speed F-actin cosedimentation assays with Vt, MVt, MVt-T978R, MVt-Tr, and MVt-Tr-T978R proteins comparing the fraction of F-actin present in bundles or in higher-order assemblies. Error bars represent S.D. of eight data points (represented as *solid* circles within the bar graph) from three independent experiments. *D*, results from high speed lipid cosedimentation assays comparing Vt, MVt, MVt-Tr bound to LUVs. Error bars in (*B,C*) represent S.D. of eight data points from two independent experiments. One-way ANOVA followed by Tukey *post hoc* performed for multiple comparisons of protein–PIP_2_ interaction in (*D*). Means of variants that do not share letter (inlet alphabet) are significantly different.

The MVcn isoform contains a 68-residue insert within the tail domain that negatively regulates actin bundling (24, 25). To evaluate the specific contribution of the MVt 68-residue insert to differential PIP_2_ association, we generated a truncated MVt variant (MVt-Tr, residues 947–1134) (Fig. 3*A*) that is equivalent in size to Vt when measured from the C-terminus. This MVt variant retains the helix 1′ and N-Strap' structural elements from the MVcn-specific 68-residue insert while lacking the remaining portions of the insert. We then conducted circular dichroism (CD) analyses and confirmed that MVt-Tr retains secondary structure and stability comparable to WT MVt (Fig. S2, *A* and *B*). Additionally, high-speed actin cosedimentation assays demonstrated that MVt-Tr maintained F-actin binding similar to MVt (Fig. 3*B*). Consistent with our previous findings (25), the low-speed actin cosedimentation assays revealed that unlike WT MVt, the MVt-Tr variant bundled F-actin similar to WT Vt (Fig. 3*C*), suggesting that the absence of the 68-residue insert restores this function. Intriguingly, lipid cosedimentation assays with MVt-Tr revealed substantially enhanced PIP_2_ association compared to WT MVt (Fig. 3*D*). Specifically, MVt-Tr exhibited a PIP_2_ association profile more closely resembling that of Vt, with the percentage of protein pelleted increasing by approximately 15% in liposomes containing 10% PS/10% PIP_2_ and by 28% in liposomes containing 20% PIP_2_ compared to WT MVt. These results provide direct experimental evidence that the 68-residue insert significantly contributes to the reduced PIP_2_-liposome association observed for MVt compared to Vt.

The 68-residue insert is undetectable in X-ray and cryo-EM structures of MVt and MVcn, likely due to it dynamic nature. Hence, structural information is lacking. Moreover, sequence analysis reveals an enrichment of acidic residues, yielding a net negative charge of −8 at physiological pH. This charge profile, along with the insert's size, likely plays a role in regulating MVt membrane association. Our experimental data with MVt-Tr strongly supports the hypothesis that the insert imposes both steric and electrostatic constraints that reduce efficient clustering of MVt at PIP_2_-enriched membranes, contributing to the reduced binding potential observed despite K_d_ values similar to Vt.

### Sequence differences in the Vt and MVt basic collar regions contribute to differential PIP_2_ recognition

To further probe the molecular basis for the reduced binding capacity of MVt with PIP_2_-containing liposomes compared to Vt, we employed a comprehensive approach combining computational methods, site-directed mutagenesis, and lipid interaction assays. Based on sequence alignment (Fig. 1*B*), we noted that Vt contains arginine at position 910 (R910) while MVt has threonine at the corresponding position 978 (T978) in the basic collar region. Our previous findings demonstrated that this basic collar region which comprises basic residues from helices 1 to 2 and the C-terminal loop plays a critical role in PIP_2_ head group recognition (34). The T978 substitution in MVt in place of the positively charged R910 in Vt suggests a potential variation that might contribute to differential PIP_2_ association.

To test our hypothesis, we generated an MVt T978R variant which contains an arginine residue at position 978 (Fig. 3*A*). We verified through far-ultraviolet CD (Fig. S2*A*) and CD thermal melt experiments (Fig. S2*B*) that MVt T978R retains secondary structure and stability relative to WT MVt (Fig. S2, *A* and *B*). Further, to assess whether MVt T978R retains actin-binding and actin-bundling properties, we performed actin cosedimentation assays (24, 25, 41). High-speed actin binding cosedimentation assays conducted using WT Vt as a positive control (Fig. 3*B*) showed that MVt-T978R exhibits similar F-actin binding and bundling compared to MVt (Fig. 3, *B* and *C*). These observations corroborate our previous findings (25) that the presence of H1 promotes the formation of a protruding subdomain structure that sterically occludes Vt dimerization and F-actin bundle formation. The protruding subdomain is not formed in MVt-Tr as it lacks H1, leading to the restoration of F-actin bundling properties. Notably, sequence differences of H1′ and N-strap′ of MVt do not affect F-actin interactions. We then conducted lipid cosedimentation assays to assess MVt T978R association with PIP_2_ liposomes. As shown in Figure 3*D*, we find that MVt T978R exhibits enhanced PIP_2_-liposome association relative to WT MVt, consistent with our computational analyses. The fraction of MVt T978R pelleted in liposomes containing either 10% PS+10% PIP_2_ or 20% PIP_2_ liposomes significantly increases by 11% and 18%, respectively, relative to WT MVt. While association is enhanced, this single MVt T978R mutation shows reduced lipid association compared to WT Vt (Fig. 3*D*). When the T978R mutation is introduced into MVt-Tr, the resulting MVt-Tr T978R variant restores PIP_2_ association to levels comparable to WT Vt (Fig. 3*D*), suggesting that both the 68-residue insert and the T978/R978 difference contribute to differential association of MVt to PIP_2_. These results indicate that reduced PIP_2_ association observed for MVt relative to Vt is due to sequence differences within the 68-residue insert.

In parallel, we performed molecular docking of full-length PIP_2_ to Vt and MVt using HADDOCK (42) (High Ambiguity Driven biomolecular DOCKing). HADDOCK uses an energy-based scoring function to predict the most favorable protein-ligand binding modes by generating and ranking potential complexes based on interaction energies. The docking studies focused on the basic collar region of Vt/MVt with PIP_2_, as we have previously shown this region to be crucial for specific recognition of PIP_2_ by Vt. The basic collar encompasses residues within helix-1 (896–911 of Vt and 964–979 of MVt), helix-2 (918–938 of Vt and 986–1006 of MVt), and the C-terminus (1047–1066 of Vt and 1115–1134 of MVt) and is enriched in basic amino acids. To improve the accuracy of PIP_2_ association predictions, the acidic N-terminal strap of Vt was omitted during the docking simulations, as its removal promotes the release of the N-terminal strap, mimicking Vcn activation and enhancing PIP_2_ association (37).

**Figure 4:**
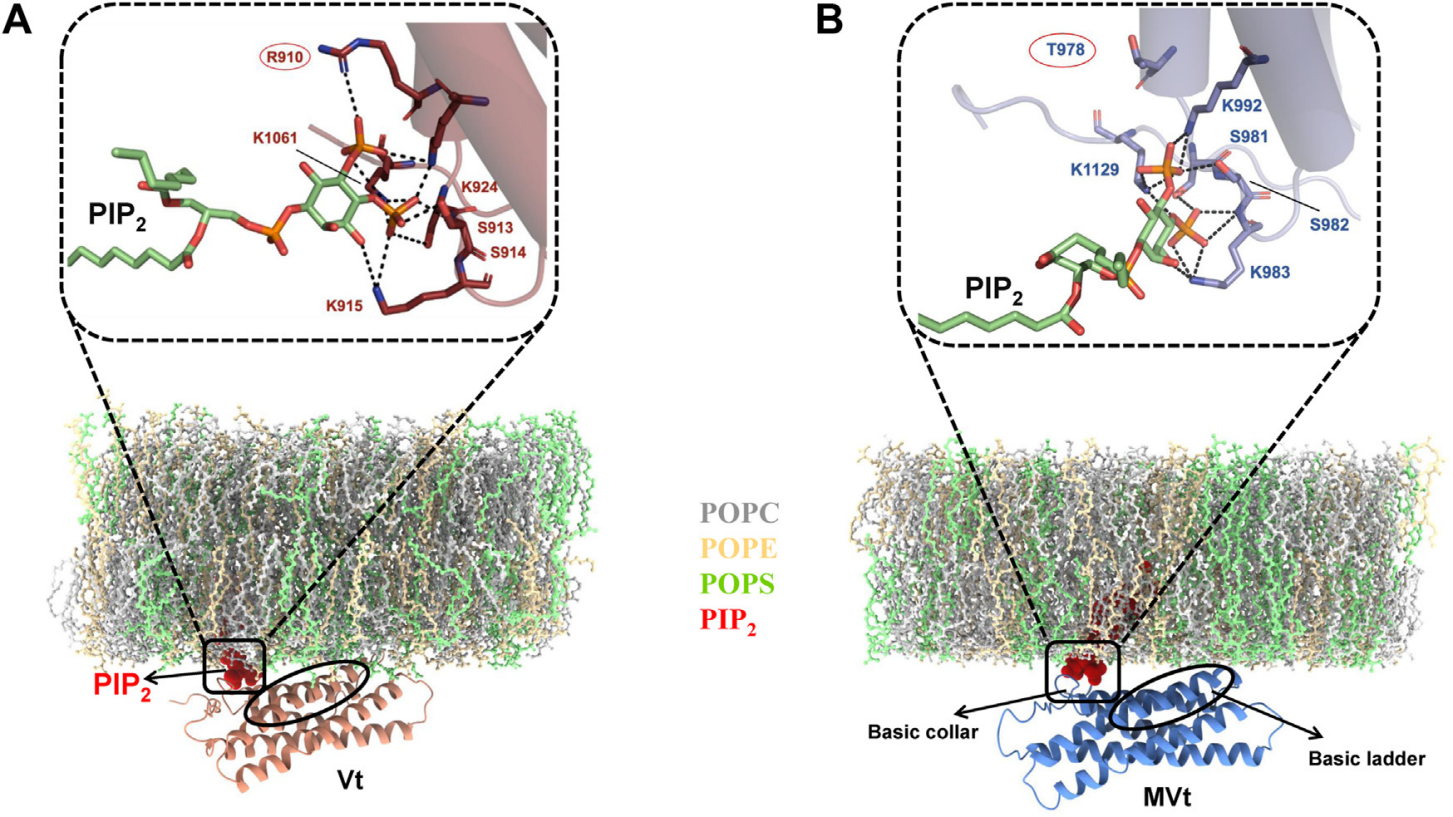
**Molecular dynamics simulations of Vt and MVt associated with PIP_2_-containing lipid bilayer reveal distinct roles of basic collar and basic ladder regions.** Snapshots from GROMACS simulations of (*A*) Vt and (*B*) MVt associated with a PIP_2_-containing lipid bilayer. The basic collar region (helices 1 and 2) of each protein engages the PIP_2_ head group (*red*), whereas the basic ladder region inserts into the lipid bilayer through interactions with POPS (*green*). The lipid bilayer components are depicted as follows: POPC (*gray*), POPE (*wheat*), and POPS (*green*) in ball and stick representation, PIP_2_ (*red*) shown as spheres, Vt (*brown*) and MVt (*blue*) proteins represented in cartoon form. Zoomed-in views illustrating PIP_2_ head group interactions with Vt (*brown*, *A*) and MVt (*blue*, *B*). Differential interactions are highlighted in each panel, with the PIP_2_ head group interactions depicted in *black* dotted lines.

Analysis of the HADDOCK docking poses revealed that in Vt, residues R910, S913, S914, K915, K924, and K1061 within the basic collar form key interactions with the PIP_2_ head group (Fig. 4*A*). Specifically, S914 interacts with the PIP_2_ C1 hydroxyl and the C3-phosphate, while K924 engages with the C1 hydroxyl and the C2 phosphate. Additionally, R910, K915, and S913 form H-bond interactions with the phosphoryl groups on C2, C6, and C3, respectively (Table 3). These observations are consistent with previous findings that the basic collar region, including residues R910, S913, S914, K915, K924, and K1061, play a critical role in PIP_2_ head group recognition (34).

**Table 3:** Comparison of residue interactions with PIP_2_ head group from computational models of Vt-PIP_2_ and MVt-PIP_2_ head group

Vt Residue (atom)	Distance (Å)	MVt residue (atom)	Distance (Å)
SER 913 (N)	2.68	SER 981 (OG)	2.59
SER 914 (N)	3.17	SER 982 (N)	2.68
SER 914 (OG)	2.56	SER 982 (N)	3.17
LYS 915 (N)	2.71	SER 982 (OG)	2.56
LYS 915 (NZ)	2.52	LYS 983 (N)	2.71
LYS 915 (NZ)	2.78	LYS 983 (NZ)	2.52
LYS 924 (NZ)	2.42	LYS 983 (NZ)	2.78
LYS 924 (NZ)	2.58	LYS 992 (NZ)	2.42
LYS 1061 (NZ)	2.65	LYS 1129 (NZ)	2.44
LYS 1061 (NZ)	2.80	LYS 1129 (NZ)	2.80
LYS 1061 (NZ)	2.72		
ARG 910 (NH2)	2.90		

We also investigated interactions of PIP_2_ with MVt by analyzing HADDOCK docking poses. The docking results revealed that residues S981, S982, K983, K992, and K1129 within the basic collar region exhibit specificity for the PIP_2_ head group. Notably, S981 interacts with the PIP_2_ C1 hydroxyl and the C3 phosphate, S982 with the C6 phosphate, and K992 with the C2 phosphate (Fig. 4*B*). Additionally, K1129 forms H-bond interactions with the C3 phosphate. Our comparative analyses between MVt and Vt predicted differences in PIP_2_ head group association due to sequence variations. For instance, Vt residue R910 forms strong H-bond interactions with the phosphoryl groups of C2, while the corresponding T978 residue in MVt does not interact with PIP_2_. Moreover, Vt residue K915 makes van der Waals interactions with the C6 phosphate, whereas the corresponding K983 residue in MVt forms H-bond interactions with the C6 phosphate (Fig. 4*B*). These observations provide strong evidence that key sequence variations in the basic collar region, particularly the R910/T978 substitution, contribute significantly to the differential PIP_2_ recognition observed between Vt and MVt, supporting our experimental findings with the MVt T978R variant.

### Sequence differences in the MVt and Vt acidic N-strap region contribute to differences in association with PIP_2_-liposomes

**Figure 5:**
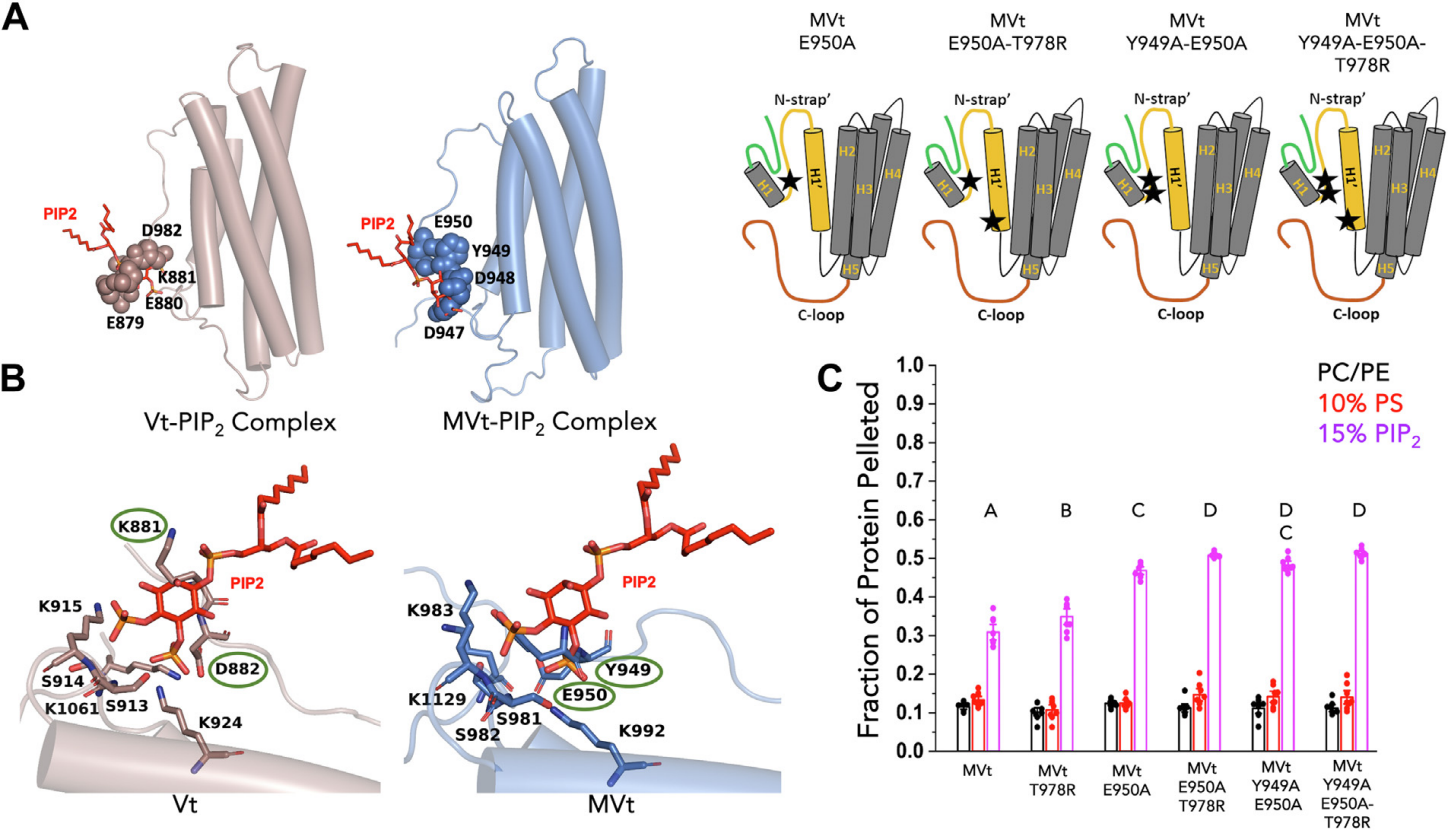
**The MVt N-terminal strap obscures association with PIP_2_-containing LUVs.***A*, overview of Vt (*left*) and MVt (*right*) showing the steric clashes between the N-terminal strap and PIP_2_ (*red*). *B*, close-up view of PIP_2_-binding pocket highlighting steric clashes (*green* circles) between the N-terminal strap residues and the PIP_2_ headgroup (stick representation). *C*, Lipid cosedimentation assays measuring the association of Vt, MVt, and MVt-Tr with LUVs as quantified by protein fractions present in pellets. Error bars in (*C*) represent the S.D. of eight data points from two independent experiments. One-way ANOVA followed by Tukey *post hoc* was performed for multiple comparisons of protein–PIP_2_ interaction. The means of variants that do not share the same letter (inlet alphabet) are significantly different.

In the autoinhibited state of full-length Vcn (PDB: 1RKE) (43), the Vt acidic N-strap region interacts with both the head domain and the basic collar region, providing electrostatic shielding of PIP_2_ association (Fig. 5*A*) (10, 12, 44). Upon release from autoinhibition, the N-terminal strap is displaced, exposing the PIP_2_-binding site and enabling PIP_2_ association with the basic collar region (37, 44). Previous studies have shown that removing the N-terminal strap from Vt significantly enhances PIP_2_ association, underscoring its regulatory role in PIP_2_ recognition (34).

Consistent with these findings, structural alignment of PIP_2_-bound Vt and MVt reveals that in the autoinhibited state, steric clashes occur between the PIP_2_ head group and residues of the N-terminal strap, leading to competitive binding with the basic collar region. Specifically, residues K881 and D882 in Vt and Y949 and E950 in MVt obstruct PIP_2_ interaction (Fig. 5*B*), further supporting the role of the N-terminal strap in modulating PIP_2_ accessibility.

To experimentally verify this electrostatic shielding effect in the context of MVt, we introduced alanine substitutions at residues Y949 and E950, creating single E950A, double Y949A-E950A, E950A-T978R, and triple Y949A-E950A-T978R variants of MVt. We then conducted CD analyses and lipid cosedimentation assays to evaluate the effect of these MVt substitutions on the structure, stability, and lipid association properties. Far-UV CD and thermal CD melt profiling of these variants indicate that alanine substitutions have minimal impact on the conformation and stability of the MVt variants relative to WT MVt (Fig. S2, *A* and *B*). All MVt N-strap′ variants show enhanced PIP_2_ association, yet to various degrees relative to Vt (Vt saturation point of 15%), as assessed by lipid cosedimentation assays (Fig. 5*C*). While the single MVt E950A and the double MVt Y949A-E950A variants show enhanced MVt-PIP_2_ association relative to T978R alone, association is still slightly lower relative to WT Vt. Of note, the N-strap′ MVt variants, when combined with the T978R substitution (E950A-T978R and Y949A-E950A-T978R), show enhanced PIP_2_ association at similar levels compared to WT Vt (Fig. 5*C*). These observations indicate that sequences differences in both the basic collar and N-strap′ of MVt and Vt contribute to differences in PIP_2_-mediated membrane association.

**Figure 6:**
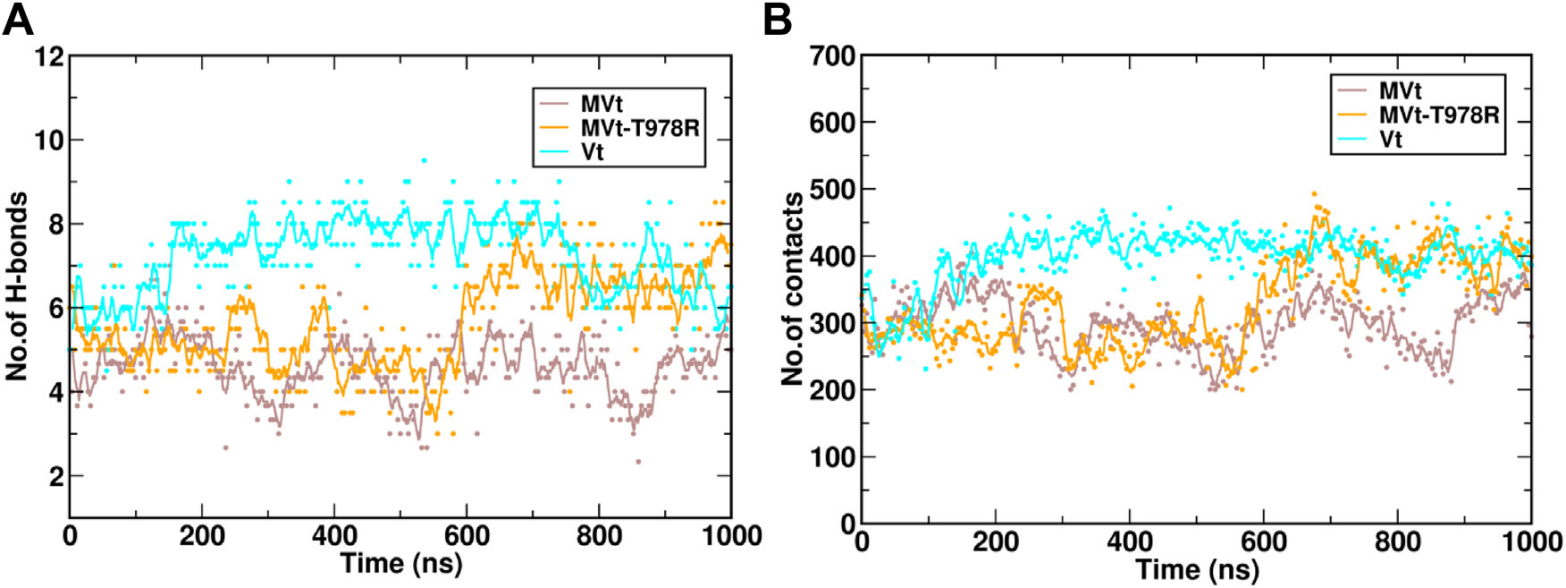
**Average number of H-bonds and nonbonded contacts between protein variants and PIP_2_ in PIP_2_-containing lipid bilayer over 1000 ns MD simulations.***A*, average number of H-bonds formed between PIP_2_ and protein variants (MVt: *brown*, MVt-T978R: *orange*, Vt: *cyan*) across triplicate 1000 ns simulations conducted in lipid bilayers containing embedded PIP_2_. SDs across replicates are shown as *dots*. *B*, total nonbonded contacts (van der Waals and electrostatic interactions) between the protein variants and PIP_2_.

### Molecular dynamics simulations reveal PIP_2_'s role in differential membrane association of Vt and MVt

To examine differential interactions between Vt and MVt with PIP_2_ membranes, we performed MD simulations focusing on the basic collar and basic ladder regions as key anchoring sites for membrane association. Given the importance of membrane composition in modulating protein–lipid interactions, we modeled a physiologically relevant lipid bilayer consisting of POPE (phosphatidylethanolamine), POPC (palmitoyl-oleoylphosphatidylcholine), and POPS (phosphatidylserine) in a 3:1:1 ratio. The MD simulations were performed for 1000 ns using the HADDOCK-derived PIP_2_-bound structures of Vt and MVt as starting conformations (Fig. 4).

To verify the structural stability of PIP_2_-protein-membrane complexes and confirm proper PIP_2_ insertion and protein anchoring, radial distribution functions (RDFs) were calculated for key bilayer components across Vt, MVt, and MVt-T978R systems (Fig. S3). The acyl chains of PIP_2_ displayed pronounced RDF peaks at distances < 20 Å in all systems, aligning with the distribution of acyl chains from POPC, POPE, and POPS. This supports full embedding of PIP_2_’s hydrophobic tails within the bilayer core, indicating proper integration.

The glycerol-ester regions of PIP_2_ exhibited distinct RDF peaks between 15 to 25 Å, matching the distribution of glycerol-ester groups in surrounding lipids (Fig. S3). In MVt-T978R, a slightly broader peak (18–25 Å) compared to Vt and MVt hints at increased flexibility in this region, likely due to the R978 mutation. PIP_2_ headgroups showed RDF peaks at distances > 25 Å, consistent with solvent-exposed positioning similar to POPS and other headgroups (Fig. S3). This distribution confirms that PIP_2_’s negatively charged headgroup remains solvent-exposed at the membrane surface, interacting with the basic collar regions of Vt and MVt. Additionally, RDFs of protein positions relative to the bilayer center revealed consistent localization > 30 Å across systems, verifying stable membrane association at the membrane interface (Fig. S3).

Next, we assessed the structural stability of each system by monitoring the RMSD of the protein backbone. Throughout the simulations, all systems remained structurally stable, with backbone RMSD values ranging from approximately 1.0 to 3.0 Å (Fig. S4*A*), indicating that Vt and MVt do not undergo significant conformational drift. Additionally, we evaluated the stability of the bound PIP_2_ molecules by measuring the RMSD of their heavy atoms. Across all systems, PIP_2_ stabilized at an average RMSD of 4.0 Å (Fig. S4*B*), suggesting a consistent mode of association. Further, analysis of secondary structure elements revealed that they remained stable throughout the simulation trajectory, further supporting the structural integrity of both proteins (Fig. S5).

Consistent with our previous model of Vt/PIP_2_ membrane association (34), the basic collar region of both proteins facilitates membrane association by interacting with the PIP_2_ head group whereas the basic ladder region (residues from helix-3 (943–971 for Vt and 1011–1039 for MVt) and helix-4 (977–1004 for Vt and 1045–1072 for MVt)) cause partial insertion into the lipid bilayer. This insertion is driven by electrostatic interactions between basic residues within these helices (K944, R945, K952, K956, R963, K966, K970, R978, R1008, and R1049 in Vt and K1012, R1013, K1020, K1024, R1031, K1034, K1038, R1046, R1076, and R1117 in MVt) and the acidic POPS head groups.

### MD simulations highlight enhanced PIP_2_-membrane association for Vt and MVt-T978R compared to MVt

Comparative analysis of MD trajectories of Vt and MVt embedded in a PIP_2_-containing lipid bilayer revealed differences in protein–PIP_2_ interactions. The sequence variation at residue 978 significantly influenced PIP_2_ engagement, affecting both direct protein–PIP_2_ interactions and overall membrane association. Specifically, the presence of the charged R910 side chain in Vt facilitates additional electrostatic interactions (7 ± 2 H-bonds, 389 ± 72 contacts) with the negatively charged PIP_2_ headgroup and POPS lipids relative to T978 in MVt (4 ± 2 H-bonds and 282 ± 96 non-bonded contacts) (Figs. 6 and S6) resulting in a stronger association of Vt relative to MVt with the PIP_2_ bilayer.

To probe the functional impact of this residue, we introduced the T978R mutation into MVt using the PyMOL mutagenesis tool. Remarkably, the mutation substantially enhanced PIP_2_ interactions with MVt-T978R (6 ± 2 H-bonds and 343 ± 106 contacts) (Figs. 6 and S6). Hence, the introduction of an arginine at position 978 partially restores electrostatic contacts with PIP_2_, promoting increased local membrane engagement, although this does not necessarily imply a direct increase in binding affinity.

### MVt alters PIP_2_-liposome association in the presence of Vt

We have previously shown that MVt negatively regulates Vt-mediated F-actin bundling relative to Vt alone and postulated that MVcn tunes Vcn-mediated actin cytoskeletal assemblies when co-expressed in muscles cells (24, 25). As MVcn is co-expressed with Vcn in muscle, we sought to evaluate whether addition of MVt alters Vt association with PIP_2_ liposomes by performing lipid cosedimentation assays using a 1:1 M ratio of MVt and Vt. Notably, the addition of MVt and MVtp elicited a slight reduction in Vt-PIP_2_ association compared to Vt alone (Fig. S7*A*), with the amount of Vt pelleted with 10% PS + 10% PIP_2_ decreased by 18% when MVt/MVtp was present at a 1:1 M ratio (Fig. S7*A*). However, the association of MVt/MVtp with PIP_2_ liposomes when Vt was present (1:1 ratio) was unaffected compared to WT MVt/MVtp alone (Fig. S7*B*). While these findings show a small reduction in Vt association with PIP_2_ liposomes at stoichiometric levels of MVt, at physiological MVcn levels, an even smaller reduction (<10%) of Vt associated with PIP_2_ membranes is likely. Next, we investigated the influence of MVt CM mutations on Vt-PIP_2_ association. When Vt was mixed with CM mutants at a 1:1 M ratio, the association of Vt with PIP_2_ was reduced to levels similar to those observed with WT MVt and MVtp (Fig. S7*C*), while the percent association of the CM MVt mutants with PIP_2_ liposomes in the presence of Vt was unaffected and similar to MVt CM mutants alone (Fig. S7*D*). Collectively, these data indicate that the MVt CM mutations do not significantly impact Vt-PIP_2_ membrane association compared to WT MVt. Therefore, based on these findings, we hypothesize that the physiological impact of MVt CM mutations is primarily due to alterations in actin binding rather than membrane association.

## Discussion

Vcn and its splice variant MVcn are key cell adhesion proteins that mediate cell–cell and cell–matrix interactions to control cell morphology, motility, and mechanotransduction. The formation and dynamic regulation of adhesion sites depend on the recruitment of various components to the membrane and are facilitated by the signaling lipid PIP_2_. Interactions of PIP_2_ with its targets play crucial roles in both the assembly of nascent adhesions (by recruiting diverse precomplex components) and in their subsequent disassembly or maturation in response to mechanical cues that transmit force across biological membranes (45, 46, 47, 48). We have previously shown that Vcn association with PIP_2_ drives recruitment and activation of Vcn at FAs as well as FA turnover (38). However, it remains unclear whether MVcn specifically associates with PIP_2_-containing membranes and how such interactions might influence its functional interplay with Vcn in muscle tissues where both isoforms coexist (49).

While a structure of MVt with a short-chain PIP_2_ mimetic is available (36), we sought to experimentally evaluate MVt interactions with full-length PIP_2_ in the context of physiological membrane components. Consistent with previous studies (19, 39), we find that MVt exhibits a weaker association for liposomes containing PIP_2_ when compared to Vt, suggesting that a smaller fraction of MVcn likely interacts with membranes in cells. Through comprehensive structural, computational, and biochemical analyses, we identified three key determinants that contribute to this differential association.

First, the 68-residue insert unique to MVt plays the most significant role in limiting membrane association, as removal of the insert in the MVt-Tr variant dramatically enhances PIP_2_-binding capacity while retaining proper MVt fold and actin-binding properties. We propose that the dynamic insert imposes steric constraints that hinder neighboring MVt molecules from efficiently clustering at the membrane surface. Second, in addition to steric effects, the N-strap′ region within the insert of MVt is enriched in acidic residues relative to N-strap of Vt, creating a net negative charge of −8 at physiological pH. This charge distribution likely generates electrostatic repulsion that further inhibits MVt clustering by preventing close molecular packing at membrane interfaces. Our experiments with N-strap variants confirm that this region contributes significantly to reduced PIP_2_ association through enhanced electrostatic shielding of the binding pocket. Third, sequence differences in the basic collar regions between Vcn and MVcn mediate specific PIP_2_ recognition. Most notably, the substitution of arginine at position 910 in Vt with threonine (T978) in MVt results in loss of critical electrostatic interactions with the PIP_2_ head group. Consistent with our experimentally directed model (34), substitution of threonine 978 in MVt with arginine results in enhanced association with PIP_2_-containing liposomes.

These combined mechanisms explain our observation that MVt exhibits lower binding potential despite similar K_d_ values compared to Vt. Individual MVt molecules can bind with comparable affinity, but the insert restricts the maximum number of molecules that can simultaneously engage the membrane, limiting overall occupancy without compromising individual binding strengths (50). This phenomenon of similar binding affinities but different binding potentials has been observed across multiple protein systems. Perhaps most relevant to our work are the key cell-cell adhesion proteins, αE-catenin and αT-catenin, that show similar affinities but different binding potentials for F-actin (51) directly paralleling our findings with Vt and MVt. Similarly, isoform-specific differences in binding capacity despite similar individual affinities have been documented in 14-3-3 protein family members that regulate cellular function (52). This phenomenon extends to other systems as well. Integrin isoforms α5β1 and αvβ3 exhibit similar binding affinities for fibronectin but significantly different maximum binding capacities, attributed to distinct conformational states that affect their clustering behavior and membrane organization (53). Moreover, AEBP2 protein isoforms, while sharing DNA-binding motifs, show different binding capacities due to structural variations that influence their ability to form higher-order complexes (54).

The differential binding potential between Vt and MVt, driven by these sequence and charge differences, likely serves as a regulatory mechanism to fine-tune the relative distribution of these proteins between membrane and cytoskeletal pools, ultimately affecting force transmission and mechanical responses in muscle tissue. Given previous findings that PIP_2_ association with the tail domain of Vcn competes with F-actin binding (17, 35), differential membrane affinity may alter the subcellular localizations for these isoforms in muscle. Notably, at physiological concentrations of PIP_2_ (1–2%), MVt shows little association with PIP_2_ liposome. Super-resolution microscopy has revealed that Vcn populates three distinct locations: the membrane/integrin signaling layer, the force transduction layer, and the actin regulatory layer (49). Based on our findings, we predict that MVt may localize to the force transduction and actin regulatory layers, preferentially engaging F-actin over the membrane. It is also possible that MVt associates with PIP_2_ membranes when the local PIP_2_ concentration increases or when clustering occurs during a signaling event (55, 56, 57).

This differential localization of these isoforms has important functional implications for adhesion dynamics and force transmission. While nascent adhesions can disassemble at the onset of force transmission (58, 59), early adhesions formed by talin–Vcn precomplexes have a higher rate of maturation (38). Therefore, PIP_2_-assisted Vcn membrane association may stabilize and could enhance the number of precomplexes in early stages of adhesion formation (49). Conversely, a higher population of MVcn associated with the cytoskeleton may alter actin reorganization by negatively regulating Vcn function during a force transmission event (24, 25). The differential membrane affinity between Vt and MVt may dictate the balance between membrane- and cytoskeleton-associated populations of these proteins, ultimately influencing adhesion morphology, dynamics, and force transmission in muscle cells. Taken together, our findings suggest that although MVcn participate in certain adhesion processes, its reduced membrane engagement compared to Vcn allows for specialized functional contributions to cellular mechanics.

Significantly, we find that MVcn CM mutations do not alter MVt liposome association, indicating that the CM pathology arising from MVcn mutations may be a consequence of their defect in actin reorganization rather than lipid binding. Interestingly, while the R975W MVt CM mutant was previously shown to possess a higher affinity for PIP_2_, we find similar association of this mutant relative to WT MVt in PIP_2_-containing LUVs. These findings suggest that the physiological impact of CM mutations on heart function may result from the impairment of actin cytoskeletal organization rather than membrane association.

In conclusion, this study elucidates molecular differences in PIP_2_–membrane interactions between Vt and MVt, providing insights into how co-expression of these isoforms in muscle may coordinately regulate FA dynamics and force transduction. Our findings demonstrate three major factors contributing to differential PIP_2_ association: (1) the presence of the 68-residue insert in MVt that likely imposes both steric and electrostatic constraints on membrane association; (2) differences in the acidic N-terminal strap that provide electrostatic shielding of the PIP_2_-binding site; and (3) sequence differences in the basic collar region, particularly the T978/R978 variation. Taken together, the findings enhance our understanding of how Vcn isoforms are differentially regulated and distributed between membrane and cytoskeletal pools in muscle cells, with important implications for understanding both normal muscle function and pathological states associated with CMs.

## Experimental procedures

### Construct design and mutagenesis

The gene fragment comprising the vinculin tail domain (Vt) residues 879 to 1066 from the chicken sequence was cloned into pQlinkH vector (Addgene). The tail domain of metavinculin (MVt) containing residues 879 to 1134 from the human sequence was cloned into 2HR-T vector (Addgene). The MVtp variant (spans residues 858-1134 and includes the proline linker that connects the MVcn head and tail domains) was commercially prepared by Genscript on 2HR-T MVt vector backbone. The MVt-Tr variant (residues 947–1134) is designed to be equivalent in length to Vt when measured from the C-terminus. This construct strategically preserves the helix 1′ and N-Strap′ regions of MVt while removing the majority of the 68-residue insert, creating a chimeric protein that maintains the critical structural elements of the MVt tail domain in a framework comparable to Vt. This design allows direct comparison of tail domain functions without the confounding effects of the full insert. MVt-Tr constructs were commercially prepared by GenScript using a 2HR-T MVt template. The MVtp CM (A934A, ΔL954, and R975W) and all the other MVt variants including T978R, E950A, E950A-T978R, Y949A-E950A, and Y949A-E950A-T978R were constructed through mutagenesis using the 2HR-T MVt plasmid backbone, appropriate primers (Eton Biosciences) and a Q5 site-directed mutagenesis kit (New England Biolabs). All constructs were verified by DNA sequencing (Eton Biosciences).

### Expression and protein purification

Vt and MVt proteins were expressed and purified as described previously (24). All expression vectors containing an N-terminal His-tag and TEV cleavage site sequence were transformed into *Escherichia coli* BL21 (DE3). The cells were first grown at 37 °C to an optical density of 0.6 to 0.8 at 600 nm, then IPTG (0.5 mM) was added to induce protein expression. Cells were grown overnight at 18 °C and harvested by centrifugation at 4500 rpm for 30 min. Vt and MVt pellets were resuspended in lysis buffer (20 mM Tris, 150 mM NaCl, 5 mM imidazole, 2 mM β-mercaptoethanol, pH 7.5 for Vt and 50 mM Tris, 200 mM NaCl, 10 mM imidazole, 2 mM β-mercaptoethanol, pH 8.0 for MVt/MVtp) (24) and purification initiated by affinity separation using Ni-NTA-agarose beads (Qiagen). Proteins bound to the beads were washed with wash buffer (20 mM Tris, 150 mM NaCl, 60 mM imidazole, 2 mM beta-mercaptoethanol, pH 7.5) before eluting the target proteins using elution buffer (20 mM Tris, 150 mM NaCl, 500 mM imidazole, 2 mM β-mercaptoethanol, pH 7.5). The eluant was dialyzed into TEV cleavage buffer (20 mM Tris, 150 mM NaCl, 50 mM imidazole, 2 mM β -mercaptoethanol, pH 7.5) overnight at 4 °C in the presence of TEV protease to remove the His-tag. Cleaved proteins were collected by running the dialyzed/cleaved solutions over Ni-NTA-agarose beads. The proteins were further purified by size-exclusion chromatography (S75 column; GE) in gel filtration buffer (10 mM Tris, 200 mM KCl, 10 mM imidazole, 2.5 mM MgCl_2_, 1 mM EGTA, 2 mM DTT, pH 7.5). Protein purity > 96% by SDS-PAGE gel densitometry was verified before further analyses. Purified proteins were concentrated by centrifugation, aliquoted, and snap-frozen using liquid nitrogen. Protein stocks were stored at −80 °C.

### CD experiments

Far-ultraviolet (260–190 nm) CD spectra were collected on WT and mutant MVt proteins to evaluate secondary structure using a Jasco J-815 CD spectrophotometer. All spectra were acquired at 25 °C in a buffer containing 10 mM potassium phosphate, 50 mM Na_2_SO_4_, and 1 mM DTT, pH 7.5. Each sample was placed in a 0.1 mm path-length (400 μl) cuvette, with spectra recorded with 0.1 nm data pitch at a scanning speed of 50 nm/min. To assess thermal stability, CD spectra were recorded at 222 nm over a temperature range of 20 to 95 °C using 1 °C intervals. CD data collection was obtained using 20 μM protein with the resultant spectra averaged over three scans.

### Lipid cosedimentation assays

To study the affinity and specific interaction of Vt, MVt, and MVt variants with acidic phospholipids, lipid cosedimentation assays were conducted using LUVs containing a subset of the following lipids: 1,2-dioleoyl-*sn*-glycero-3-phosphocholine (PC), 1,2-dioleoyl-*sn*-glycero-3-phosphoethanolamine (PE), 1,2-dioleoyl-*sn*-glycero-3-phospho-L-serine (PS), and L-α-phosphatidylinositol-4,5-bisphosphate (PIP_2_, brain, porcine) from Avanti Polar Lipids. Four different LUVs of varying composition were used to evaluate binding interactions and phospholipid specificity of MVt proteins: 1) polar uncharged LUVs composed of PE/PC at a 3:2 ratio, 2) charged acidic phospholipid LUVs composed of PE/PC/PS at a 3:1:1 ratio, 3) charged acidic phospholipids with 10% PIP_2_ comprised of PE/PC/PS/PIP_2_ at a 3:1:0.5:0.5 ratio, and 4) charged acidic phospholipids with 20% PIP_2_ composed of PE/PC/PIP_2_ at a 3:1:1 ratio. To quantify the capacity of the membrane vesicles for association with Vt, MVt, and the MVt A934 CM mutant with PIP_2_-containing membranes, cosedimentation experiments in PE, PC and PS containing LUVs with varying PIP_2_ concentrations (0–30 mol%) were performed. The lipid combination was mixed, dried under nitrogen glass, and left in a vacuum overnight as previously described (60, 61). Dried lipids were hydrated in buffer (40 mM Hepes, 150 mM NaCl, 2 mM DTT, pH 7.4) for 2 h, with constant shaking, and then extruded with 100 nm polycarbonate membranes in a mini-extruder (Avanti) to form LUVs. LUVs were stored overnight at 4 °C. The following day, 250 μg of lipid and 10 μl of 100 μM protein (in an identical buffer) were added to each vesicle sample, producing a final volume of 100 μl and incubated for 1 h at RT. The lipid–protein mixture was spun at 100,000*g* in a Beckman TLA100 rotor for 1 h at RT. Supernatants and pellets were run on a 15% SDS-PAGE gel, stained with Coomassie Brilliant Blue, and analyzed by ImageJ Software (62).

### Actin cosedimentation assays

To assess whether MVt variants predicted to alter PIP_2_ interactions affect F-actin binding and higher-order F-actin assembly, we conducted actin cosedimentation assays under conditions similar to methods reported previously (61). Briefly, monomeric actin (G-actin) was purified from rabbit muscle acetone powder (obtained from Pel-Freez Biologicals) and stored at −80 °C in storage buffer (50 mM imidazole, 100 mM NaCl, 10 mM MgCl_2_, 10 mM EGTA, 0.5 mM DTT, 0.2 mM ATP, pH 7.0). Polymerization of G-actin (100 μM) to F-actin was achieved by incubation in actin polymerization buffer (10 mM Tris, 200 mM KCl, 10 mM imidazole, 2.5 mM MgCl_2_, 1 mM EGTA, 2 mM DTT, pH 7.5) at room temperature under slow constant rotation for 30 min. For both F-actin binding and bundling analyses, 100 μl samples were prepared by mixing 10 μM MVt and MVt variant proteins in actin polymerization buffer and 20 μM F-actin. Samples were then incubated at room temperature for 1 h and centrifuged. Actin-binding assays were conducted using high-speed cosedimentation assays (185,000 RCF for 1 h) whereas actin bundling was quantified by a low-speed centrifugation at 12,000 RCF for 12 min. The fraction of MVt proteins interacting with F-actin in the pellet and supernatant was quantified by SDS-PAGE electrophoresis. Densitometry was performed using ImageJ (62).

### Molecular docking of Vt, MVt, and MVt-T978R with PIP_2_

Vt (residues 895–1065, PDB: 1QKR) and MVt (residues 959–1134, PDB: 3MYI) were employed as starting points for the simulations. To prepare the protein structures for docking, bound water molecules from the crystal structures of Vt and MVt were removed. Additionally, the Vt and MVt N-strap region was removed from the structures prior to docking, based on previous findings that removal of the N-strap enhances PIP_2_ association without compromising the overall protein structure. Missing residues and atoms were modeled using homology modeling *via* the Modeller-9v21 program (63). Forty models were generated, and the conformer with the lowest Modeler Objective Function (m.o.f) score was selected for subsequent studies. To generate the MVt-T978R variant structure, the PyMOL mutagenesis tool (64) was used to substitute threonine for arginine at position 978 in the MVt homology model. Subsequently, sidechain conformations of R978 were optimized to maximize interactions with neighboring residues while minimizing perturbations to the backbone structure.

We then employed HADDOCK (42) (High Ambiguity Driven protein-protein DOCKing) to predict PIP_2_ interactions with Vt, MVt, and MVt-T978R. The PIP_2_ structure (1,2-dioleoyl-sn-glycero-3-phospho-(1′-myo-inositol-4′,5′-bisphosphate)) was parameterized with the sn-1 chain as oleate (C18:1) and the sn-2 chain as arachidonate (C20:4). The inositol headgroup was modeled in its fully ionized state (charge: −4), consistent with physiological pH. Ambiguous interaction restraints were defined based on the basic collar regions of Vt/MVt (lysine/arginine-rich residues in Vt and in MVt) and the phosphate groups of PIP_2_. HADDOCK’s three-stage protocol included rigid-body docking (1000 poses), semi-flexible refinement (T = 300 K, 200 cycles), and explicit solvent refinement (water, 8 Å shell). The final poses were ranked using HADDOCK’s energy score (weighted sum of van der Waals, electrostatic, and desolvation energies). The top-ranked pose for each system, exhibiting optimal headgroup coordination with the basic collar and minimal steric clashes, was selected for membrane insertion.

### Molecular dynamics simulations of Vt, MVt, and MVt-T978R embedded in a lipid bilayer

To elucidate the molecular basis underlying the differential association of MVt and Vt with PIP_2_-containing liposomes, we conducted MD simulations. These simulations were designed to assess the stability of the protein–lipid complexes, investigate structural differences, and explore membrane interactions. CHARMM-GUI (65) was employed incorporating a physiological lipid composition of PE, palmitoyl-oleoylphosphatidylcholine (PC), and PS in a 3:1:1 ratio, respectively. The HADDOCK-derived docking poses, representing the best binding modes of PIP_2_-bound Vt, MVt, and MVt-T978R, were then embedded on the lipid bilayer such that the hydrophobic tails of the PIP_2_ molecules were positioned within the bilayer, while the PIP_2_ headgroups interacted with the basic collar regions of the proteins. Subsequently, the systems were solvated using the TIP3P water model. To prevent simulation artifacts, any trapped water molecules located within the hydrophobic core of the lipid bilayer were identified and removed using a custom Perl script. MD simulations were carried out using GROMACS-2021.5 (66, 67) with the CHARMM36 (68) force field with energy minimization (steepest descent, 5000 steps) followed by a 10 ns equilibration phase under NVT (303.15 K, V-rescale thermostat) and NPT (1 bar, Parrinello-Rahman barostat) ensembles. Positional restraints on protein and lipid heavy atoms were gradually relaxed (force constants: 1000 → 100 → 10 → 0 kJ/mol·nm^2^). Nonbonded interactions were computed with a van der Waals cutoff of 12 Å and an electrostatic cutoff of 12 Å, and periodic boundary conditions were applied in all three dimensions to facilitate comprehensive sampling of the conformational space and interactions between PIP_2_ and the proteins. Replicates were generated by rebuilding systems in CHARMM-GUI (65) with distinct random seeds for initial velocities to enhance conformational sampling. This approach helps to account for variability in the simulation outcomes due to the initial conditions. Each simulation run was performed for a duration of 1000 ns, allowing for sufficient sampling of the conformational space of the Vt, MVt, and MVt-T978R proteins embedded in the lipid bilayer.

### Analysis of MD simulations trajectories

To analyze the MD simulation trajectories, several analyses were performed to assess the stability and interactions of the PIP_2_-bound protein complexes. RMSD of the protein backbone atoms and PIP_2_ heavy atoms were calculated using *gmx rms* tool to monitor the overall stability and conformational changes of Vt, MVt, and MVt-T978R throughout the simulations. The H-bond analysis was performed using *gmx hbond* with a donor–acceptor distance cutoff of 3.5 Å and a hydrogen–donor–acceptor angle cutoff of 30°. Nonbonded interactions between the protein and lipid bilayer were analyzed using the *gmx mindist* tool with a 5 Å cutoff. The tool was employed to calculate the minimum distances between the protein's backbone and sidechain heavy atoms and the lipid's headgroup and glycerol-ester atoms. Secondary structure was determined using the *gmx do_dssp* tool, which applies the standard DSSP (Define Secondary Structure of Proteins) algorithm. This method classifies secondary structure elements—such as α-helices, β-sheets, turns, and coils—based on hydrogen bonding patterns and backbone dihedral angles. RDFs between lipid components (PIP_2_ headgroups, glycerol-ester linkages, and acyl chains) and the protein were calculated using the *gmx rdf* tool. RDFs were computed across the Vt, MVt, and MVt-T978R systems to assess spatial distributions and potential interactions of PIP_2_ relative to the protein and surrounding membrane lipids (POPC, POPE, POPS). RDFs were centered on the protein’s center of mass or relevant residue groups, and distributions of PIP_2_ subgroups (acyl tails, glycerol backbones, and headgroups) were analyzed relative to both the protein and membrane components. Standard RDF parameters were used with a maximum cutoff of 50 Å and a bin width of 0.1 Å. *XMGrace* (69) and inhouse scripts were employed to generate detailed graphs and plots from simulation data. For rendering high-quality images and creating informative movies from the computational data, both PyMOL (64) and ChimeraX (70) were employed.

### Statistical analysis

Experimental data sets were statistically compared using *t* test. An unpaired 2-tailed *t* test was performed when comparing two means. All data were presented as mean ± S.E.M. unless otherwise noted. Values for *N*, *n*, *p*, and statistical test performed are listed in the figure captions. Statistical significance was set at ∗*p* < 0.05; ∗∗*p* < 0.01; ∗∗∗*p* < 0.001; *p* > 0.05, not significant (n.s.). One-way ANOVA followed by *post hoc* Tukey (equal variances) or *post hoc* Steel-Dwass test (unequal variances) of Vt, MVt, and MVt variant interactions with F-actin and PIP_2_ was performed for multiple comparisons using Student Version of Origin software (OriginLab Corporation, MA-01060). Group multiple comparisons of means are represented with inlet alphabets within the figures. Means of variants that do not share the same letter (inlet alphabet) are significantly different. Kinetic data were analyzed using nonlinear regression in GraphPad Prism, fitting to a one-site–specific binding model to determine rate constants (K_d_, B_max_, and B_p_).

## Data availability

All data are contained within the manuscript and supporting information. The structures used in this study were accessed from the Protein Data Bank (PDB). Molecular dynamics simulation data are available from the corresponding author upon reasonable request. Any plasmids and recombinant proteins described in this study are available from the corresponding author upon reasonable request subject to a completed Materials Transfer Agreement.

## Supporting information

This article contains supporting information.

## Conflict of interest

The authors declare that they have no conflicts of interests with the contents of this article.
