# Supplementary material for: Molecular basis for differential PIP2-mediated association between vinculin and its splice isoform metavinculin
Source: J Biol Chem. 2025 May 14;301(6):110232. doi: 10.1016/j.jbc.2025.110232 (PMC12180986; doi:10.1016/j.jbc.2025.110232)
Supplement: Supporting Information Figures [file mmc1.pdf]

Suppl. Fig.1

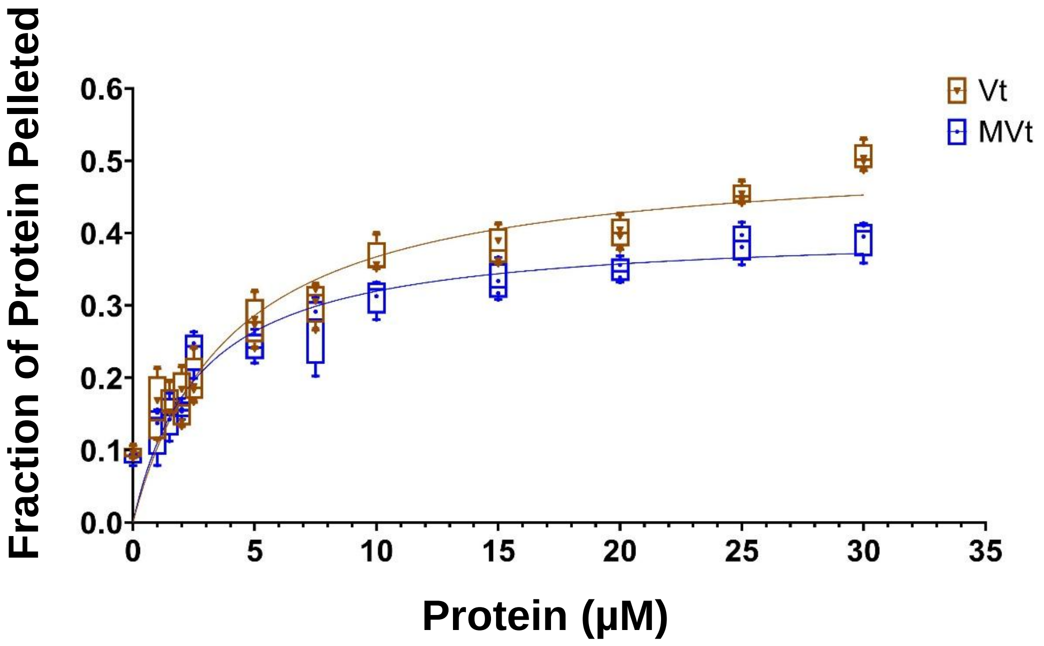
Fraction of Protein Pelleted
0.0
0.1
0.2
0.3
0.4
0.5
0.6
0
5
10
15
20
25
30
35
Protein (µM)
Vt
MVt

Suppl. Fig.2

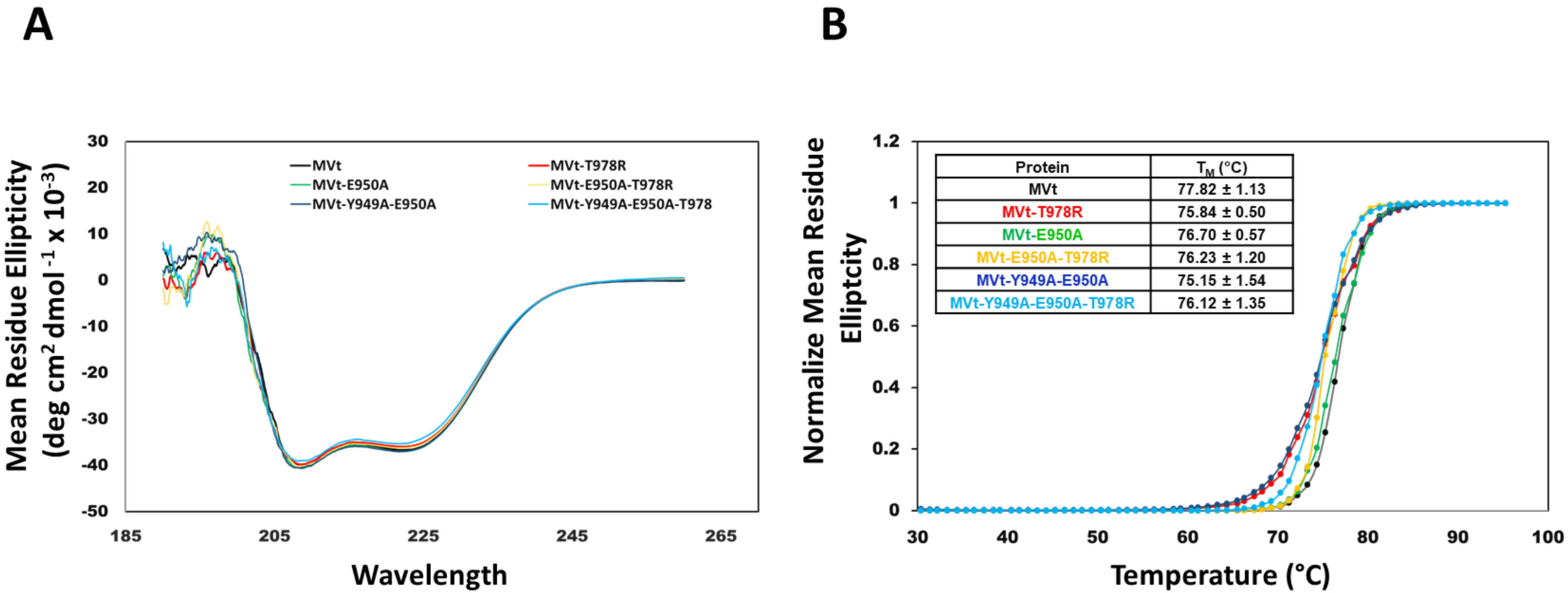
A
Mean Residue Ellipticity (deg cm2 dmol -1 x 10-3)
Wavelength
MVt
MVt-T978R
MVt-E950A
MVt-E950A-T978R
MVt-Y949A-E950A
MVt-Y949A-E950A-T978
B
Normalize Mean Residue Ellipticity
Temperature (°C)
Protein | TM (°C)
MVt | 77.82 ± 1.13
MVt-T978R | 75.84 ± 0.50
MVt-E950A | 76.70 ± 0.57
MVt-E950A-T978R | 76.23 ± 1.20
MVt-Y949A-E950A | 75.15 ± 1.54
MVt-Y949A-E950A-T978R | 76.12 ± 1.35

Suppl. Fig.3

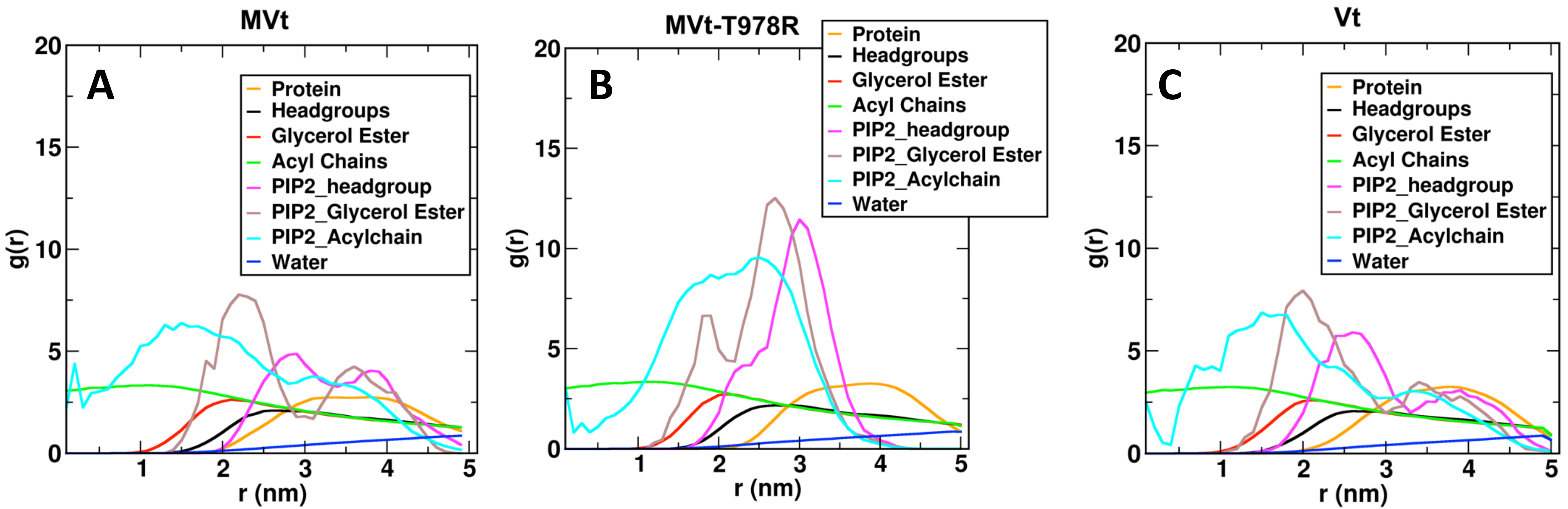
MVt
A
MVt-T978R
B
Vt
C
Protein
Headgroups
Glycerol Ester
Acyl Chains
PIP2_headgroup
PIP2_Glycerol Ester
PIP2_Acylchain
Water
g(r)
r (nm)
0
5
10
15
20
1
2
3
4
5

Suppl. Fig.4

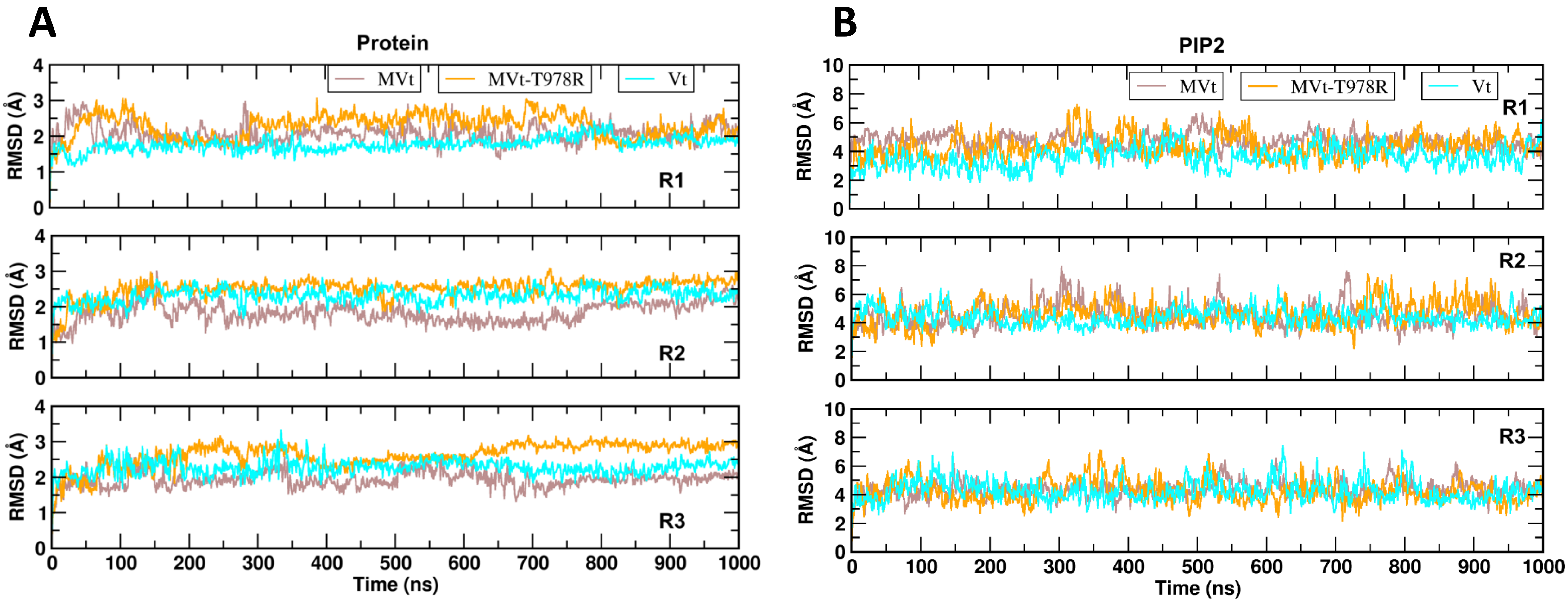

A
Protein
MVt
MVt-T978R
Vt
RMSD (Å)
R1
R2
R3
Time (ns)
B
PIP2
MVt
MVt-T978R
Vt
RMSD (Å)
R1
R2
R3
Time (ns)

Suppl. Fig.5

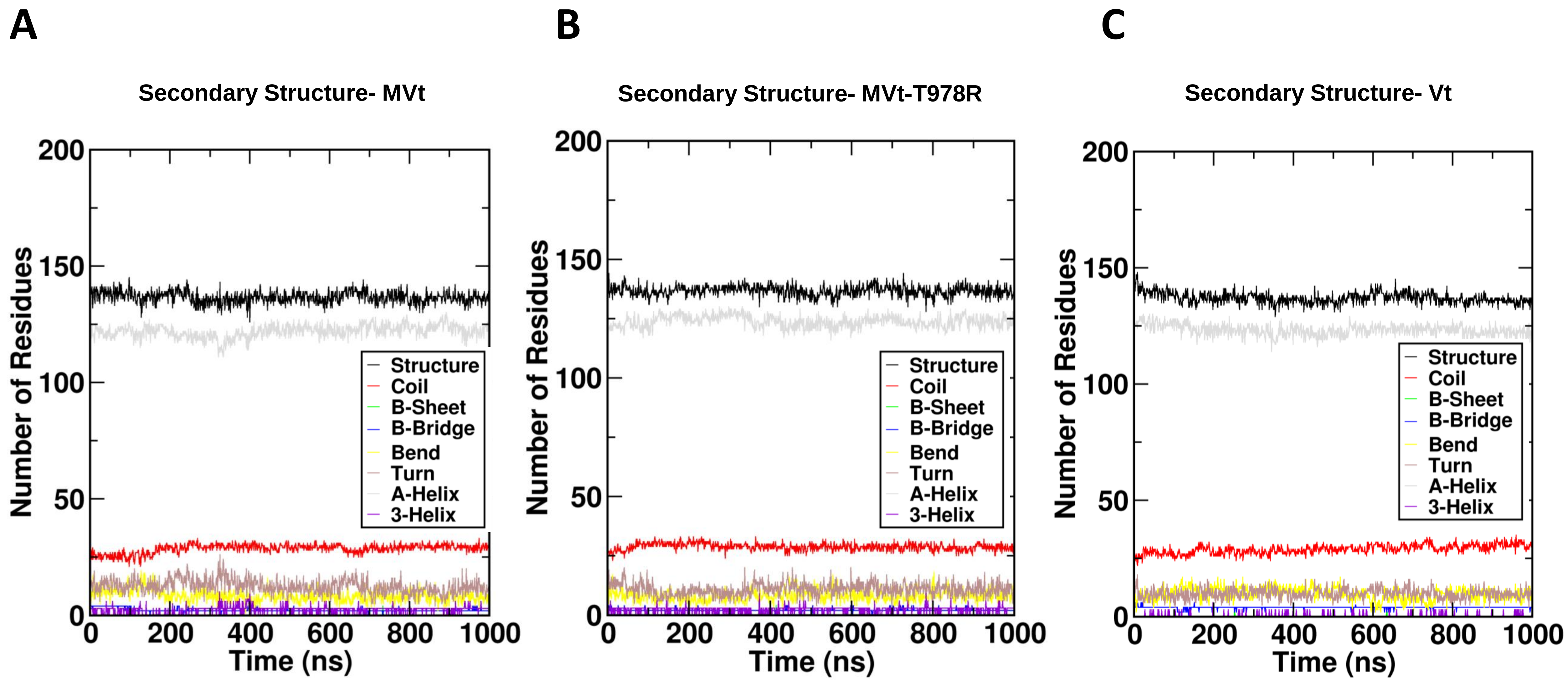
A
Secondary Structure- MVt
Number of Residues
Time (ns)
Structure
Coil
B-Sheet
B-Bridge
Bend
Turn
A-Helix
3-Helix
B
Secondary Structure- MVt-T978R
Number of Residues
Time (ns)
Structure
Coil
B-Sheet
B-Bridge
Bend
Turn
A-Helix
3-Helix
C
Secondary Structure- Vt
Number of Residues
Time (ns)
Structure
Coil
B-Sheet
B-Bridge
Bend
Turn
A-Helix
3-Helix

Suppl. Fig.6

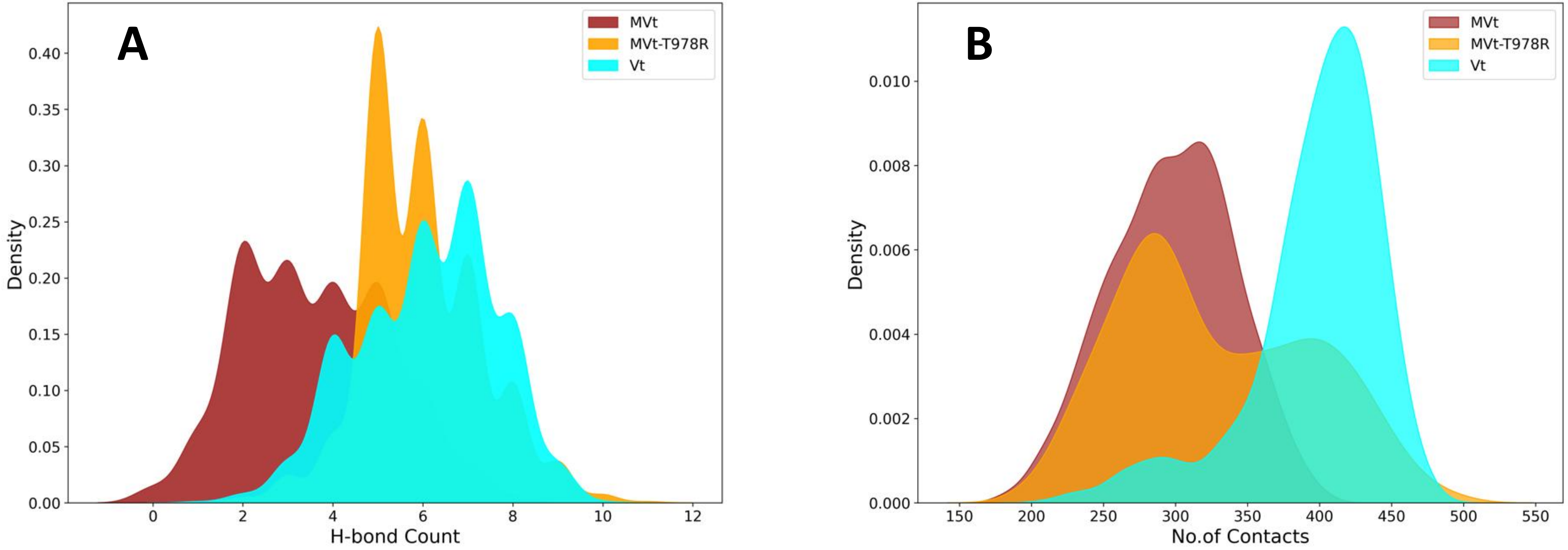
A
MVt
MVt-T978R
Vt
Density
H-bond Count
0.00
0.05
0.10
0.15
0.20
0.25
0.30
0.35
0.40
0
2
4
6
8
10
12
B
MVt
MVt-T978R
Vt
Density
No.of Contacts
0.000
0.002
0.004
0.006
0.008
0.010
150
200
250
300
350
400
450
500
550

Suppl. Fig.7

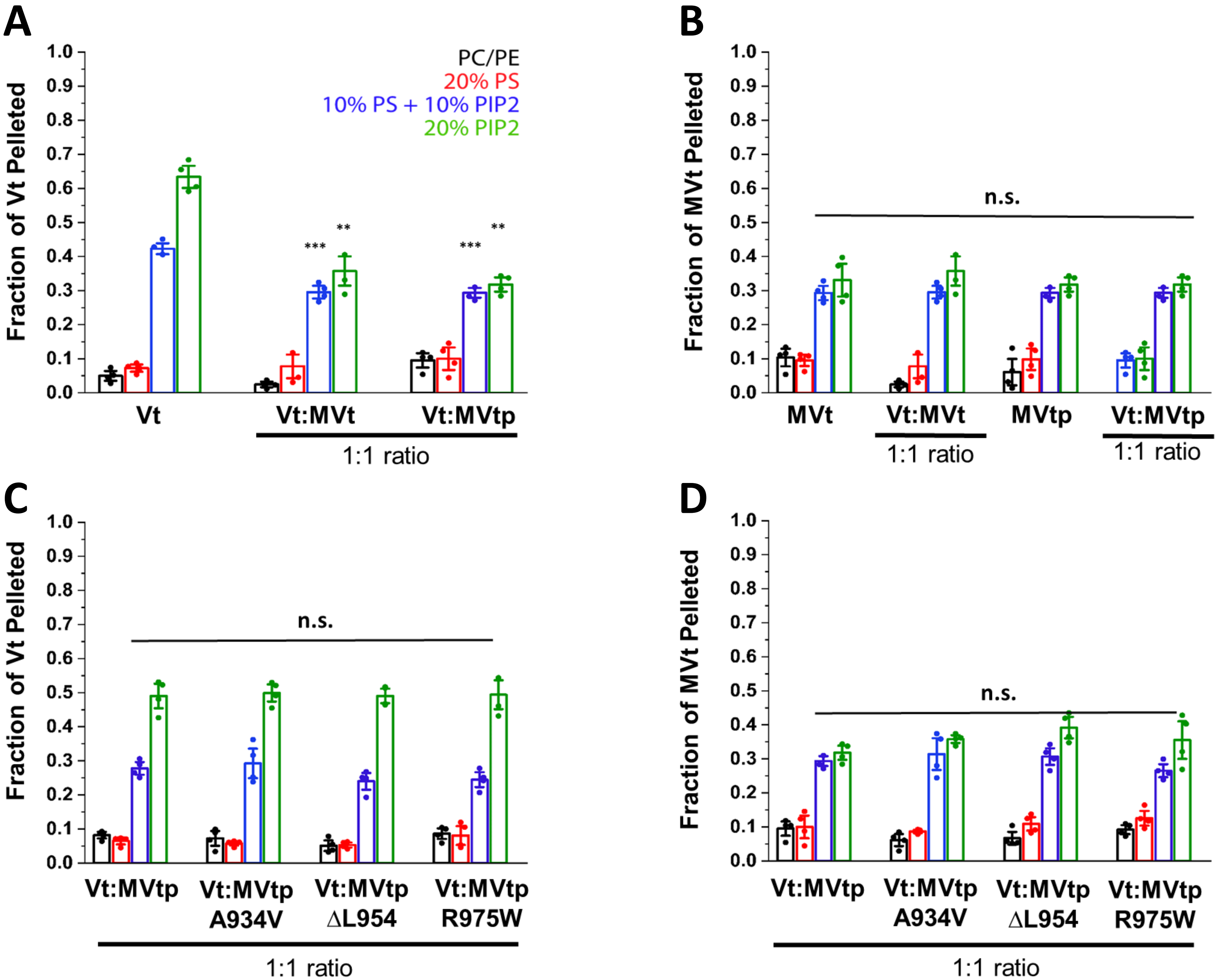
A
Fraction of Vt Pelleted
PC/PE
20% PS
10% PS + 10% PIP2
20% PIP2
***
**
Vt
Vt:MVt
Vt:MVtp
1:1 ratio
B
Fraction of MVt Pelleted
n.s.
MVt
Vt:MVt
MVtp
Vt:MVtp
1:1 ratio
1:1 ratio
C
Fraction of Vt Pelleted
n.s.
Vt:MVtp
Vt:MVtp A934V
Vt:MVtp ΔL954
Vt:MVtp R975W
1:1 ratio
D
Fraction of MVt Pelleted
n.s.
Vt:MVtp
Vt:MVtp A934V
Vt:MVtp ΔL954
Vt:MVtp R975W
1:1 ratio

Suppl. Fig.8

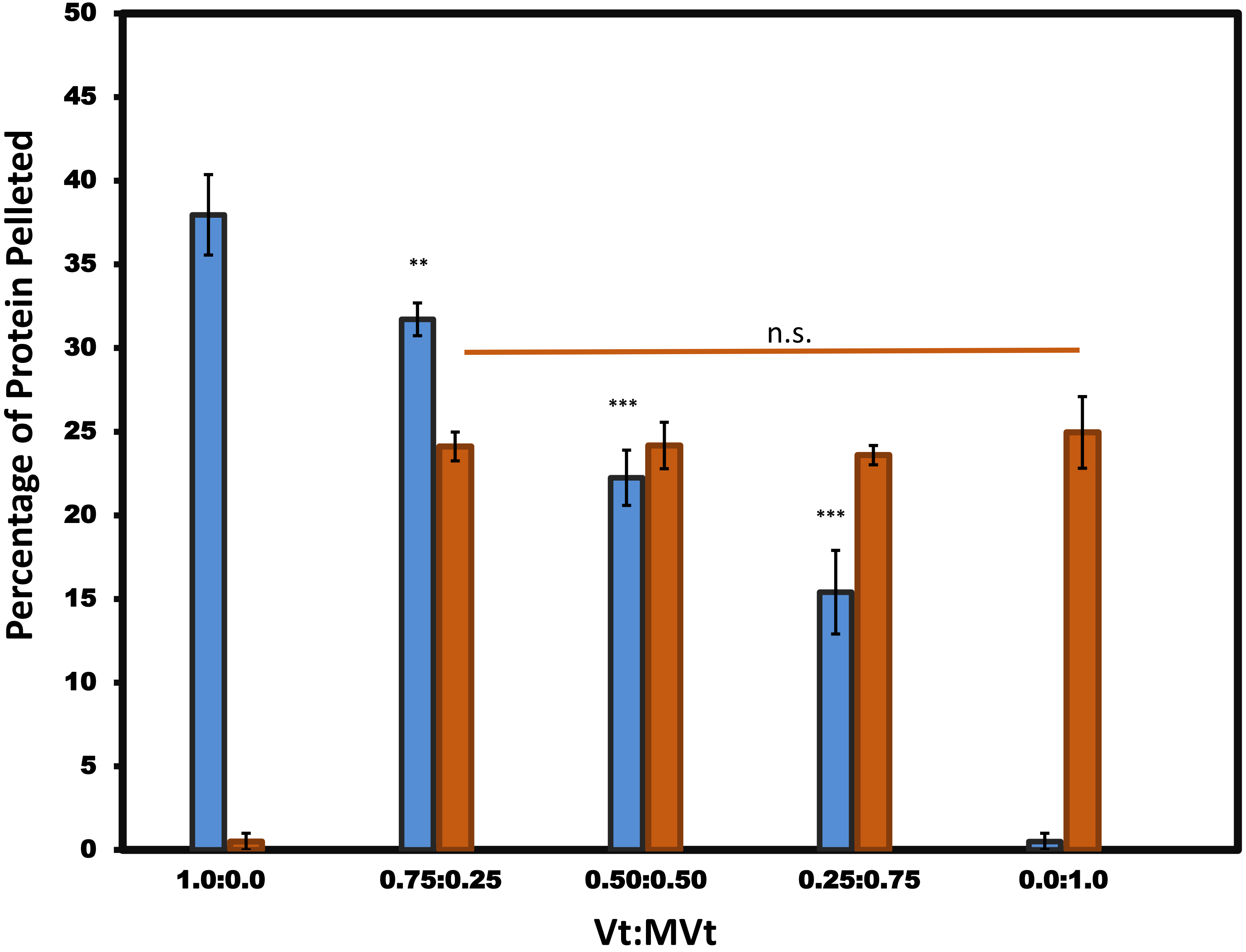

50
45
40
35
30
25
20
15
10
5
0
Percentage of Protein Pelleted
**
***
***
n.s.
1.0:0.0
0.75:0.25
0.50:0.50
0.25:0.75
0.0:1.0
Vt:MVt
