# Supplementary material for: Molecular basis for differential PIP2-mediated association between vinculin and its splice isoform metavinculin
Source: J Biol Chem. 2025 May 14;301(6):110232. doi: 10.1016/j.jbc.2025.110232 (PMC12180986; doi:10.1016/j.jbc.2025.110232)
Supplement: Supporting Information [file mmc2.docx]

**Supplemental Fig. 1. Protein titration experiments reveal differential Vt and MVt association with PIP_2_-containing liposomes.** High-speed lipid co-sedimentation assays examining concentration-dependent association of Vt and MVt to PIP_2_-containing liposomes. Increasing concentrations of proteins (0-30 μM) were incubated with simplified PC/ PIP_2_ (9:1) liposomes to specifically isolate interactions between the basic collar region and PIP_2_ headgroups. Binding curves were fitted using nonlinear regression to a one-site specific binding model, yielding apparent binding affinity (K_d_) and maximum binding capacity (B_max_) values reported in Table 2. Individual data points are represented within the box & whisker plot with appropriate bars showing range and mean. This targeted approach reveals that the MVt 68-amino acid insert fundamentally alters PIP_2_ headgroup recognition independent of other membrane components.

**Supplemental Fig. 2. MVt T978R variant and N-terminal strap residue substitutions retain MVt structure and stability. A)** Far-UV circular dichroism (CD) spectral profiles of WT MVt and MVt variants. **B)** Melting temperature (T_M_) of WT and MVt variants calculated from CD thermal melt curves. Representative CD data are shown from three independent experiments. The T_M_ melt curves are presented as an average of 3 scans from 3 independent experiments. Error in T_M_ is shown with the standard errors.

**Supplemental Fig. 3. Radial Distribution Functions (RDFs) of key bilayer components and protein in PIP_2_ containing lipid bilayer over 1000 ns MD simulations.** Radial distribution plots showing the spatial organization of key bilayer components relative to the protein in simulations performed with PIP_2_-containing membranes for **A)** MVt, **B)** MVt-T978R, and **C)** Vt. The RDFs were calculated from the final 500 ns of each MD trajectory. Sharp peaks observed in the PIP_2_ headgroup and lipid headgroup distributions indicate stable interactions with the protein, while the RDFs of acyl chains confirm proper insertion of PIP_2_ and lipid components within the membrane bilayer.

**Supplemental Fig. 4. RMSD of protein and PIP_2_ in PIP_2_-embedded membrane systems over 1,000 ns MD simulations. A)** Backbone RMSD of MVt (brown), MVt-T978R (orange), and Vt (cyan) over 1,000 ns simulations performed in lipid bilayers containing embedded PIP_2_. RMSD values were calculated using protein backbone atoms from three independent replicates (R1, R2, R3) to assess structural stability and reproducibility. **B)** RMSD of the PIP_2_ molecule relative to the bilayer computed across the same triplicate simulations. The stable PIP_2_ RMSD values confirm its consistent anchoring within the membrane environment, validating the integrity of membrane insertion across all systems.

**Supplemental Fig. 5. Secondary structure stability of MVt, MVt-T978R, and Vt across 1,000 ns simulations in PIP_2_-containing membrane.** Time-resolved secondary structure profiles of **A)** MVt, **B)** MVt-T978R, and **C)** Vt proteins extracted from 1,000 ns simulations in PIP_2_-embedded membrane systems. Secondary structure elements were analyzed using the *gmx do_dssp* tool. All three protein systems exhibit stable secondary structure profiles over time with minimal transitions, indicating overall structural stability.

**Supplemental Fig. 6. Distribution of protein-PIP_2_ H-bonds and non-bonded contacts between protein variants and PIP_2_ in PIP_2_ containing lipid bilayer over 1,000 ns MD simulations. A)** Probability distribution plots of the number of H-bonds formed between each protein and MVt variant (MVt, MVt-T978R) and Vt, and PIP_2_ molecules, calculated from the final 500 ns of each trajectory. The distributions highlight the relative differences in H-bonding profiles, with Vt and MVt-T978R exhibiting a broader range of stable interactions compared to MVt. **B)** Probability distribution of total non-bonded contacts between the proteins and PIP_2_, including van der Waals and electrostatic interactions, calculated using a 6 Å cutoff. The enhanced contact frequency in Vt and MVt-T978R systems, relative to MVt, highlights the contribution of R978 in stabilizing PIP_2_ interactions.

**Supplemental Fig. 7. PIP_2_ liposome association with Vt is reduced in the presence of MVt. MVt cardiomyopathy mutations have minimal impact on Vt association with PIP_2_ liposomes. A)** Lipid co-sedimentation of Vt in the presence of MVt/MVtp at 1:1 molar ratio. **B)** Lipid co-sedimentation of MVt/MVtp in presence of Vt at 1:1 molar ratio. **C)** Lipid co-sedimentation of Vt in presence of MVt CM variants at 1:1 molar ratio. **D)** Lipid co-sedimentation assays of CM variants in presence of Vt at 1:1 molar ratio. An unpaired 2-tailed t-test performed comparing means of MVt and MVtp to Vt in Fig. 2B. Error bars represent standard deviation (S.D.) of 4 data points (represented as solid circles within the bar graph) from 2 independent experiments. Statistical significance was set at: **, ***p*** <0.01. ***, ***p*** <0.001. n.s., not-significant.

**Supplemental Fig. 8. Addition of MVt with Vt reduces PIP_2_-liposome association.** Lipid co-sedimentation of Vt in the presence of increasing MVt concentrations. Fraction of Vt and MVt pelleted are represented in blue and brown, respectively. Vt association with PIP_2_ containing LUVs decreases with the increasing concentration of MVt. Error bars represent standard deviation (S.D.) of 6 data points (represented as solid circles within the bar graph) from 2 independent experiments. Statistical significance was set at: **, ***p*** <0.01. ***, ***p*** <0.001. n.s., not-significant.
